# Human iPSC-derived cardiac-specific extracellular matrix scaffolds for cardiomyocyte maturation and post-myocardial infarction repair

**DOI:** 10.1016/j.bioactmat.2025.06.044

**Published:** 2025-09-17

**Authors:** Dhavan Sharma, Wenkai Jia, Alvis Chiu, Hee Jae Jang, Vladislav Leonov, Zhishi Chen, Brandon Zhao, Weijia Luo, Hutomo Tanoto, Jianhua Zhang, Alexey V. Glukhov, Yong Yang, Yuxiao Zhou, Jiang Chang, Timothy J. Kamp, Feng Zhao

**Affiliations:** a Department of Biomedical Engineering, Texas A&M University, College Station, TX, 77843, USA; b Department of Medicine, University of Wisconsin-Madison, Madison, WI, 53705, USA; c Stem Cell and Regenerative Medicine Center, University of Wisconsin-Madison, Madison, WI, 53705, USA; d Institute of Biosciences and Technology, Texas A&M University, Houston, TX, 77030, USA; e Department of Mechanical Engineering, Texas A&M University, College Station, TX, 77843, USA; f Department of Biomedical Engineering, University of North Texas, Denton, 76203, TX, USA

**Keywords:** Cardiac-specific extracellular matrix, Cardiomyocyte maturation, Cardiac tissue engineering, Anisotropic scaffold, Myocardial infarction

## Abstract

Myocardial infarction (MI) remains a leading cause of heart failure due to the limited regenerative capacity of the adult myocardium. The therapeutic efficacy of current engineered cardiac patches is hindered by their simplistic scaffold composition and lack of structural organization. This study presents a bioactive, anisotropic extracellular matrix (ECM) scaffold derived from human induced pluripotent stem cell-differentiated cardiac fibroblasts (hiPSC-CF-ECM) that combines cardiac-specific proteins and growth factors with complex structural composition. Compared to primary cardiac fibroblast ECM (pri-CF-ECM) and human dermal fibroblast ECM (hDF-ECM), hiPSC-derived cardiomyocytes (hiPSC-CMs) cultured on the cardiac-specific ECM scaffold exhibited enhanced maturation, as confirmed by bulk RNA sequencing, electrophysiological mapping, and optical-based strain analysis. In an immune-competent rat MI model, the hiPSC-CF-ECM transplantation preserved cardiac function, increased ejection fraction, and reduced maladaptive remodeling. These findings highlight hiPSC-CF-ECM as a promising biomimetic scaffold for cardiac tissue engineering and MI treatment.

## Highlights

• A cardiac-specific extracellular matrix (ECM) derived from human induced pluripotent stem cell–derived cardiac fibroblasts (hiPSC-CFs) provides a physiologically relevant microenvironment.
• hiPSC-derived cardiomyocytes (hiPSC-CMs) cultured on hiPSC-CF-ECM exhibited structural and functional maturation.
• hiPSC-CF-ECM transplantation improved ejection fraction and reduced maladaptive remodeling in a rat myocardial infarction (MI) model.
• hiPSC-CF-ECM offers a donor-independent, completely biological, and physiologically relevant matrix for cardiac tissue engineering.

## Graphical abstract

**Figure:**
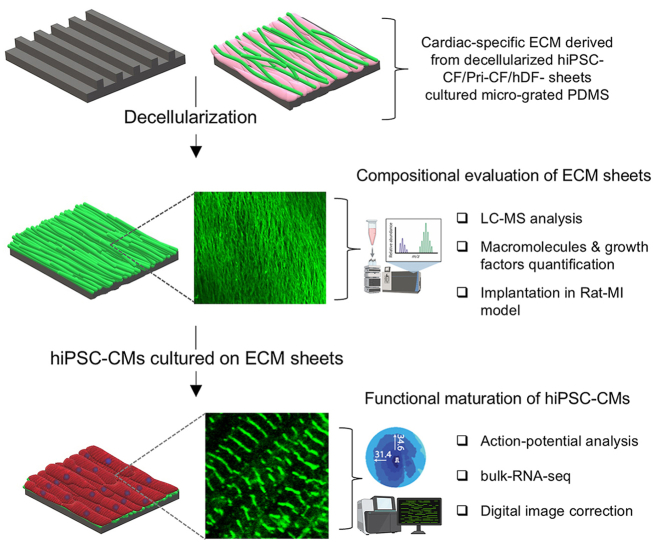

## 1 Introduction

End-stage ischemic heart failure remains a leading cause of morbidity and mortality worldwide, largely due to the limited regenerative capacity of the adult myocardium [1]. Current interventions for end-stage heart failure are limited to left ventricular assist device implantation or heart transplantation, which have major limitations and risks of life-threatening complications [[2], [3], [4]]. Recent advances in the field of human induced pluripotent stem cells (hiPSCs) offer a promising alternative to treat heart failure by differentiating hiPSCs into functional cardiomyocytes (CMs) that can be delivered to repair the failing heart [5]. In addition, previous research has devised techniques to generate large-scale, high-purity hiPSC-CMs capable of regenerating cardiac muscle in large animal models following myocardial infarction (MI) [6,7]. However, therapeutic efficacy remains limited due to the challenges in achieving graft maturation and avoiding graft-associated arrhythmias [5,[8], [9], [10], [11], [12], [13]].

To date, several strategies have been explored to enhance CM engraftment, survival, and maturation. Biomaterials such as hydrogels comprising a few major extracellular matrix (ECM) proteins that are native to hearts (e.g., collagen, fibrin, and others) have been utilized to encapsulate CMs, print 3-D tissues as cell-laden bioinks, and create electrospun fibers as scaffolds to construct cardiac tissues [9,[14], [15], [16], [17], [18], [19]]. Unfortunately, even though biologically active, these biomaterials do not fully replicate the biochemical and biophysical cues present in the native cardiac ECM, which maintains cardiac homeostasis by controlling proliferation, maturation, and differentiation of various cardiac cell types, including CMs, cardiac fibroblasts (CFs), endothelial cells and immune cells [20]. In the myocardium, the interstitial matrix — primarily composed of collagens I and III — provides mechanical support, while the pericellular matrix, rich in fibronectin, laminin, pro-collagens, hyaluronic acid, and proteoglycans, regulates the polarity, function, differentiation, and migration of cardiac cells via outside-in signaling through cell-surface receptors [21,22]. Unsurprisingly, ECM composition significantly influences its regenerative efficacy. This has been demonstrated by decellularized fetal and neonatal rat cardiac ECM, which contain more fibronectin and emillin-1 but less collagen I than adult rat ECM, with the fetal ECM promoting the most cardiomyocyte proliferation [23]. Similarly, the decellularized cardiac ECM of the myocardium-regenerating zebrafish, which has more elastin and glycosaminoglycans and less collagen than adult mouse cardiac ECM, has been shown to improve mouse heart regeneration [24]. Moreover, the cardiac ECM serves as a reservoir for ECM-bound growth factors, matrix metalloproteinases, chemokines, protease inhibitors, and micro RNAs, all of which orchestrate cardiac function in homeostasis and disease [25]. Recent studies have conclusively indicated that decellularized ECM derived from native myocardium, whether from explanted human hearts or various animal sources, improves the structural and functional maturation of hiPSC-CMs [26].

To better recapitulate the critical extracellular microenvironment, hiPSC-CMs have been co-cultured with CFs in micro-spheroids [27,28]. The hiPSC-CMs exhibited improved functional maturation and contractility via activating cyclic adenosine monophosphate pathways and forming gap junctions. However, the impact of direct CM-CF co-culture on arrhythmia development in cultured tissues remains inconclusive, as some recent studies suggest that CFs contribute to arrhythmogenesis [28], while others do not [27]. To mitigate potential CF-induced arrhythmia, hiPSC-CMs have instead been cultured with ECM particles derived from decellularized and milled human ventricular myocardium [19,29]. The hiPSC-CM displayed organized and ∼16 % longer sarcomeres and improved calcium handling compared to CMs aggregates cultured without ECM particles [19]. Similarly, pre-conditioning hiPSC-CMs with decellularized adult human left ventricular ECM has been shown to enhance differentiation, develop a mature mitochondrial network, and shift towards a more energetically efficient metabolic profile, leading to increased metabolic maturity [29]. Although promising, these approaches compromise the architectural integrity of native myocardial ECM, which is essential for anisotropically organizing CMs to achieve directed mechanical contractions [30,31]. To achieve CM alignment, previous studies have used 3-D dynamic culture stimulation or stretching of hiPSC-CMs embedded in fibrin-based hydrogels [16,32,33]. However, these methods often result in poor cell alignment or require custom stretching apparatuses. Lastly, therapeutic studies using young developmental-age human cardiac ECM, which would be more potent than adult ECM, face challenges related to scarcity and ethical concerns.

To address these challenges, we have developed a cell-derived ECM scaffold that replicates the neonatal cardiac ECM's compositional complexity and anisotropic architecture using human induced pluripotent stem cell-derived CFs (hiPSC-CFs). hiPSC-CFs provide an unlimited cell source to produce cardiac specific ECM [34,35]. Using hiPSC-CF-ECM avoids the implantation of proliferating CFs, which can lead to arrhythmic behavior depending on their activation state and abundance [27,28]. Furthermore, hiPSC-CF-ECM is not subject to the ethical limitations associated with neonatal and fetal human cardiac ECM and allows for scalable, reproducible production with minimal batch-to-batch variability. To obtain cardiac-specific ECM with anisotropic architecture, we previously optimized the culture duration of highly aligned hiPSC-CF sheets on micro-grated polydimethylsiloxane (PDMS) substrates [36] and developed a decellularization method [37] that preserves the ECM's intricate biochemical composition and architectural features. The key objective of this study is to evaluate the impact of anisotropic and cardiac-specific ECM on hiPSC-CM's maturation. Here, we compared the ECM composition from hiPSC-CF, human adult ventricular primary cardiac fibroblasts (pri-CF), and human dermal fibroblasts (hDF). Our findings revealed that hiPSC-CF-ECM promotes the structural and functional maturation of hiPSC-CMs. hiPSC-CMs cultured on these ECM scaffolds responded to the architectural cues provided by the ECM nanofibers to achieve anisotropic organization and structural maturation. Bulk RNA sequencing (RNAseq) data provided insights into the molecular mechanisms underlying the functional maturation of hiPSC-CMs cultured on hiPSC-CF-ECM, as assessed through electrophysiological and digital image correction (DIC) based strain measurement analysis. Lastly, we demonstrated the potential of hiPSC-CF-ECM alone in treating cardiac repair in a rat MI model.

## 2 Materials and methods

### 2.1 Human iPSC-CM differentiation and culture

DF19-9-11T.H iPSCs (WiCell RRID:CVCL_K054) were differentiated to CMs with an established protocol via temporal modulation of canonical Wnt signaling [7,38,39]. Briefly, the iPSCs were seeded at a density of 200,000 cells/cm^2^ on Matrigel-coated 6-well plates. The cells were cultured in mTeSR1 medium until they reached full confluence after 5–6 days, marking the start of differentiation (day 0). On day 0, the culture medium was changed to RPMI (61870036; Gibco) + B27 without insulin (1895601; Gibco) supplemented with 12 μM CHIR99021 (4423; Tocris) for 24 h. From day 1–3, the cells were maintained in RPMI + B27 without insulin. On day 3, half of the spent medium was mixed with fresh RPMI + B27 without insulin and supplemented with 5 μM IWP2 (3533; Tocris) for another 48 h. The medium was replaced with RPMI + B27 without insulin on day 5, and from day 7 onwards, it was switched to RPMI + B27 with insulin (17504044; Gibco) with feeding every other day until day 15. The purity of the differentiated iPSC-CMs was measured by flow cytometry by analyzing the cardiac Troponin T positive cells (cTnT^+^) and cryopreserved at the 15-day differentiation (Fig. S7). The hiPSC-CMs with 80–90 % purity were used for all the experiments. The iPSC-CMs were thawed on Synthemax (Corning Inc., Corning, NY) coated 6-well plates and cultured in RPMI + B27 complete supplement for 10 days. On day 25 post-differentiation, the iPSC-CMs were seeded on PDMS or glass coverslips at 180,000–210,000 cells/cm2 density. Electrophysiological optical mapping was done on day 30–35 post differentiation.

### 2.2 Human iPSC-CF differentiation and culture

DF19-9-11T.H iPSCs (WiCell RRID:CVCL_K054) were differentiated to CFs using previously published protocol [34]. Briefly, the iPSCs were seeded at a density of 200,000 cells/cm^2^ on Matrigel-coated 6-well plates. The cells were cultured in mTeSR1 medium until they reached full confluence after 5–6 days, marking the start of differentiation (day 0). On day 0, the medium was changed to 2.5 ml of RPMI + B27 without insulin, supplemented with 12 μM CHIR9902, for a 24-h treatment period. The medium was then changed to 2.5 ml of RPMI + B27 without insulin for another 24 h on day 1. Before day 3, within 24 h post-day 2, the medium was replaced with 2.5 ml of CFBM medium (containing DMEM high glucose, 500 μg/mL human serum albumin, 0.6 μM Linoleic Acid, 0.6 μg/mL Lecithin, 50 μg/mL ascorbic acid, 7.5 mM Glutamax, 1.0 μg/mL Hydrocortisone hemisuccinate, 5 μg/mL recombinant human insulin) supplemented with 75 ng/ml bFGF (WiCell Research Institute), and this feeding regimen was continued every other day until day 20 in differentiation. The 20 days differentiated iPSC-CFs were dissociated and passaged in T75 flasks (without any ECM coating) for 2 passages. The iPSC-CFs were then harvested and cryopreserved. The purity of the iPSC-CFs was measured by flow cytometry by analyzing the fibroblast marker using the TE-7 antibody (Fig. S7). The iPSC-CFs with purity above 70 % were used in the experiments.

### 2.3 Fabrication of cardiac and non-cardiac fibroblast sheets

Polydimethylsiloxane (PDMS) molds with aligned grooves were cast from micro-grated silicon wafers with 5 μm grating width, 8 μm grating pitch, and 260 nm grating depth. PDMS substrates were coated with polydopamine and type I collagen (Sigma-Aldrich, St Louis, MO) prior to cell seeding as described previously [40]. Briefly, PDMS substrates were immersed in 0.01 % W/V 3-hydroxytyramine hydrochloride (Dopamine-HCl) (ACROS Organics, Fisher Scientific) solution overnight at room temperature. PD-coated PDMS substrates were air dried and sterilized with ethylene oxide gas followed by bovine collagen coating (20 μg/mL) (Sigma Aldrich, St. Louis, MO) for 2 h [40]. Traditional PDMS treatment method involving oxygen plasma to convert silane (Si-CH3) into silanol (Si-OH) groups for collagen coating via glutaraldehyde crosslinking. However, this approach suffers from unstable surface hydrophilicity, brittle SiOx layer formation, and crack formations on the PDMS surface, which alters micro-nano scale features [41]. We have previously shown that unlike plasma treatment, PD and collagen coating significantly improves surface elastic modulus and hydrophilicity of micro-grated PDMS substrates compared to pristine PDMS, enabling better cell adhesion. Moreover, PD based coating method is simple, fast, inexpensive and avoids use of toxic chemicals such as glutaraldehyde for covalent crosslinking of collagen. [40]. Cardiac fibroblasts, including hiPSC-CFs and adult ventricular cardiac fibroblasts (Pri-CF, Lonza, Walkersville, MD) and non-cardiac fibroblasts human dermal fibroblasts (hDF) (ATCC) were seeded at a density of 20,000 cells/cm^2^ on PD and collagen coated PDMS and cultured for 2 weeks to fabricate cell sheets. All three types of fibroblasts were from passage 6. hiPSC-CFs sheets were maintained in FibroGro complete media kit (Millipore, Burlington, MA) by replacing the same volume of supplied glutamine with GlutaMAX (Lifetech) and adding 2 % FBS (R&D systems, Minneapolis MN). hiPSC-CFs sheets were maintained in Fibroblast Growth Medium-3 (FGM-3, Lonza). hDF-sheets were maintained in Dulbecco's modified Eagle's medium (DMEM) with 20 % fetal bovine serum (FBS). Culture media was changed every 48 h during the two weeks of cell-sheet culture.

### 2.4 Fabrication of cardiac and non-cardiac ECM scaffolds

Aligned hiPSC-CF, Pri-CF, and hDF sheets were decellularized to fabricate aligned hiPSC-CF-ECM, Pri-CF-ECM, and hDF-ECM, respectively, as described previously [37,42,43]. Briefly, hiPSC-CF, Pri-CF, and hDF sheets were treated with a decellularization solution-I (1M sodium chloride (NaCl), 10 mM Tris, and 5 mM ethylenediaminetetraacetate (EDTA)) for 20 min on a slow shaker, followed by three thorough washes of PBS for 10 min each at RT. Next, the cell sheets were treated with a decellularization solution-II (0.1 % Sodium dodecyl sulfate (SDS), 10 mM Tris, and 5 mM EDTA) for 40 min on a slow shaker at room temperature [37,43]. Finally, ECM sheets were thoroughly washed with PBS and incubated in FBS-containing media for 48 h on a slow shaker at RT. SDS and EDTA were used for decellularization as SDS disrupts the lipid bilayers of cell membrane and helps in releasing and solubilizing DNA while, EDTA is a chelator that binds to metallic ions to disrupt cell adhesion to ECM [37]. Prior to seeding hiPSC-CMs, decellularized ECM sheets were sterilized with 70 % v/v ethanol under UV for 30 min. After sterilization, the ECM sheets were washed twice with sterile PBS and incubated under EB20 medium overnight.

### 2.5 Quantification of key macromolecules and growth factors of ECM sheets

All three types of ECM sheets (hiPSC-CF-ECM, Pri-CF-ECM, and hDF-ECM) cultured in 24 well plates with 1.9 cm^2^ area were used to quantify ECM-bound growth factors and ECM macromolecules. Commercially available enzyme-linked immunosorbent assays (ELISA) kits (R&D Systems) were used to determine the quantity of vascular endothelial growth factor-A (VEGF), insulin-like growth factor (IGF), fibroblast growth factor-2 (FGF-2), Angiotensin II, endothelin I, and fibronectin. Elastin was quantified using the Fasting Elastin Assay Kit (Biocolor Ltd. Belfast, United Kingdom). Glycosaminoglycan (GAG) content was quantified using the Blyscan Sulfated Glycosaminoglycan Assay Kit (Biocolor Ltd.). Soluble and insoluble collagens were quantified by using Sircol Assay (Biocolor Ltd.). Every kit was used according to the manufacturers’ instructions.

### 2.6 Field emission scanning electron microscopy (FE-SEM)

ECM sheets were prepared for FE-SEM using a protocol published previously [42]. Briefly, ECM sheets were thoroughly rinsed with PBS and were fixed with 10 % formalin solution for 24 h at 4^0^C. Crosslinked samples were thoroughly washed with PBS and immersed in a series of ethanol solutions with 10 %, 30 %, 50 %, 70 %, 90 % and 100 % concentrations. Following dehydration, samples were desiccated overnight, coated with platinum-palladium alloy (Pt-Pd) using a Cressington 208 HR sputter coater (Ted Pella Inc., Redding, CA) and observed under the Hitachi S-4700 FE-SEM (Tokyo, Japan).

### 2.7 Liquid chromatography–mass spectrometry (LC-MS)-based compositional evaluation of ECM sheets

LC-MS-based compositional analysis of ECM sheets was performed by Creative Proteomics (Shirley, NY). ECM sheets from each group (hiPSC-CF-ECM, Pri-CF-ECM, and hDF-ECM) (3 repeats for each group, total 9 samples) were digested by trypsin, identified and quantified using a nano LC-MS/MS platform comprising an UltiMate™ 3000 RSLCnano System coupled with a Q Exactive HF mass spectrometer (Thermo Fisher Scientific, USA) with an ESI nanospray source. Detailed methods for sample preparation, nano LC-MS/MS analysis, and data analysis are provided in the supplementary methods document.

### 2.8 hiPSC-CM culture on cardiac and non-cardiac ECM

On day 15, CMs were seeded at a density of 1 × 10^5^ cells/cm^2^ onto the ECM sheets in EB20 medium. After 48 h, EB20 medium was replaced with RPMI/B-27+insulim medium. The hiPSC-CMs were cultured in ECM sheets for a total of 7 days by changing media at the interval of 48 h to fabricate the cardiac patch.

### 2.9 Immunofluorescence (IF) and elastin staining

ECM scaffolds were stained with primary antibodies targeting key ECM proteins and hiPSC-CMs specific proteins including collagen I, fibronectin, and laminin prepared in 1 % bovine serum albumin solution (BSA) with the ratio of 1:100. Secondary antibodies goat-anti mouse Alexa Fluor™ 488 conjugate and goat-anti rabbit Alexa Fluor™ 594 conjugate antibodies were used for IF staining. Cell nuclei were stained with 4′,6-diamidino-2-phenylindole, dihydrochloride (DAPI). Immunofluorescence and confocal images were captured using an Olympus FV-1000 confocal microscope. Elastin fibers in the ECM sheets were observed via the Verhoeff Van Gieson elastic stain kit (Polysciences, Inc., Warrington PA) following the manufacturer's information.

### 2.10 RNA isolation and bulk RNA-seq analysis

The total RNA was extracted using a RNeasy Mini Kit (Qiagen, Valencia, CA) and was treated with DNase I (Thermo Fisher) to remove genomic DNA contamination following the manufacturer's information. The bulk-RNA sequencing analysis of total RNA was performed by Novogene Inc. Downstream analysis was performed using a combination of programs, including STAR, HTseq, Cufflink, and our wrapped scripts. Alignments were parsed using the STAR program and differential expressions were determined through DESeq2/edgeR. Gene Ontology (GO) and Kyoto Encyclopedia of Genes and Genomes (KEGG) enrichment was implemented by ClusterProfiler. Gene fusion and difference of alternative splicing events were detected by Star-fusion and rMATS software. See the supplementary method document for the detailed methods.

### 2.11 Evaluating hiPSC-CMs beating rate from cardiac patch

The beating rate and maximum principal strain were calculated from each confocal video by performing DIC in DaVis software (DaVis 10.2.1, LaVision, Gottingen, Germany). DIC is an optical technique that tracks the deformation of patterned structures by performing correlation analysis between reference image (undeformed image) and deformed image. During DIC analysis, each image was divided into smaller regions (subset) and the displacement vectors of each subset between reference image and current image was calculated. In this study, videos including about 400 images (704 μm × 532 μm) were analyzed with ∼13 μm × 13 μm subset, with a step size of one-third of the subset size (overlap between consecutive subsets). Three discrete regions of interest (ROIs) with high cell densities were selected from each video and the subset with the largest deformation in each ROI was selected for further analysis. The maximum principal strain values were computed from displacement vector fields. For each subset, strain was calculated as the average over approximately 22 beating cycles. The strain values remained consistent across the beating cycles within each subset. The beating rate in each ROI was then extracted by measuring the number of periodic maximum principal strain variation cycles during a certain amount of time.

### 2.12 Electrophysiological optical mapping

Optical action potentials were captured using the FluoVolt™ membrane potential probe (F10488; Life Technologies). The staining solution contained 1 μM FluoVolt supplemented with a 1:1000 dilution of 20 % pluronic acid (P2443; Sigma-Aldrich), in Hank's Balanced Salt Solution (HBSS 14025092; Gibco). Monolayers on PDMS chips were incubated with the staining solution for 30 min in the 5 % CO_2_ incubator at 37 °C and washed out and incubated in HBSS for 10 min at 37 °C. For optical mapping, monolayers were placed in a perfusion chamber with laminar HBSS flow maintained at 37 °C throughout the experiment. Excitation light was generated by LED (X-Cite XYLIS, Excelitas Technologies, CA, USA) with an excitation filter (475/35 nm; Thorlabs, NJ, USA). The emitted light was long-pass filtered (520/35 nm; Thorlabs, NJ, USA) for the action potential signal. Emission was captured by a MiCAM Ultima-L CMOS camera (SciMedia, CA, USA) offering high spatial (100 × 100 pixels, 60 ± 10 μm/pixel) and temporal (333 frames/second) resolution. The fluorescence signals were digitized, amplified, and visualized through proprietary software (SciMedia, CA, USA). Optical signals were analyzed using a custom MATLAB-based program [44]. Monolayers underwent pacing at frequencies of 1, 2, and 3 Hz with 10-s recordings for each frequency. Stimulation was applied via bipolar platinum electrodes with a 10 ms pulse duration and a voltage amplitude ranging from 1 to 5 V, positioned close to but not in direct contact with the monolayer. Conduction velocity (CV) was measured both longitudinally (along alignment - conduction velocity longitudinal (CV_L_)) and transversely (perpendicular to the alignment - conduction velocity transverse CV_T_), with anisotropy ratio calculated as the longitudinal to transverse conduction velocity ratio (AR). Additionally, action potential durations at different percentages of repolarization were assessed.

### 2.13 Myocardial infarction induction, patch treatment, and cardiac function assessment in rats

Myocardial infarction (MI) was surgically induced in female Sprague-Dawley rats (n = 20, 9–10 weeks old, 180–210 g) following established protocols [45]. Animals were randomly allocated into four experimental groups. Prior to surgery, rats were anesthetized, and the thoracic region was shaved and disinfected with 75 % ethanol. Endotracheal intubation was performed, and mechanical ventilation was maintained under isoflurane anesthesia (2.5–3.0 %) with oxygen supplementation (1 L/min). A left thoracotomy was performed between the third and fourth ribs to expose the heart. Under microscopic guidance, the left anterior descending coronary artery (LAD) was identified and permanently ligated proximal to the first major left branch using a 6-0 polypropylene suture to induce ischemia (Fig. S6A). Sham-operated controls underwent identical procedures excluding LAD ligation.

Following the induction of infarction, an engineered cardiac patch was precisely positioned over the ischemic region to ensure complete coverage. The patch was affixed to the pericardium using three to four interrupted 8-0 polypropylene sutures (Prolene) placed 0.5–1 mm from the edges. Sutures were secured with minimal tension to prevent tissue damage while ensuring patch stability (Fig. S6B). Care was taken to avoid deep myocardial penetration to minimize arrhythmogenic risk. Final patch placement was verified to ensure secure adhesion without impeding cardiac function.

After patch implantation, the thoracic cavity was carefully closed in layers. Residual air and blood were evacuated from the pleural space prior to final closure. The muscle layer was approximated using 6-0 absorbable sutures (Vicryl), followed by skin closure with 6-0 non-absorbable monofilament sutures (Nylon). Anesthesia was gradually discontinued by reducing isoflurane concentration to 0 % while maintaining oxygen flow. Extubation was performed upon return of spontaneous respiration and toe-pinch reflex. Postoperative monitoring included continuous observation of vital signs, ambulatory recovery, and pain assessment for 3 h in a temperature-controlled recovery chamber before transferring animals to the housing facility.

### 2.14 Assessment of cardiac function with echography

Cardiac function was evaluated utilizing the Vevo 3100 imaging system (Fujifilm VisualSonics). Echocardiographic assessments were conducted at three timepoints: one day prior to surgical intervention (baseline), one-week post-surgery, and six weeks post-surgery. High-resolution B-mode and M-mode imaging of the left ventricle was acquired from the parasternal long-axis view (PSLAX) at frame rates exceeding 200 frames per second (fps) to optimize temporal resolution for precise myocardial motion analysis during the cardiac cycle. Pulsed-wave Doppler (PW) measurements of mitral valve inflow were obtained from the apical four-chamber view. Quantitative hemodynamic and structural parameters were derived using Vevo Lab analytical software. B-mode datasets were employed to compute left ventricular ejection fraction (EF) via volumetric analysis. M-mode tracings facilitated calculation of left ventricular internal diameter during systole (LVIDs) and diastole (LVIDd). PW Doppler spectra were analyzed to determine isovolumic relaxation time (IVRT) and mitral valve pressure half-time (PHT).

For GLS analysis, speckle-tracking echocardiography (STE) was performed using Vevo Strain software. The endocardial border of PSLAX B-Mode was manually traced at end-diastole, and the software automatically tracked myocardial deformation across subsequent frames. Segmental longitudinal strain curves were generated, and peak systolic GLS was calculated as the average strain of all left ventricular segments. Image quality was verified to ensure adequate tracking, and segments with poor tracking were excluded or manually adjusted.

### 2.15 Statistical analysis

Statistical comparisons between experimental groups were performed by one-way ANOVA and Tukey's post hoc test using GraphPad Prism software. Brown-Forsythe test was performed for homogeneity of variances and Quantile-Quantile plots were used for normality test. Results displayed as mean ± standard deviation and were considered statistically significant for ∗p < 0.05, ∗∗p < 0.01, ∗∗∗p < 0.001, ∗∗∗∗p < 0.0001. For optical mapping p-values were determined using one-way ANOVA followed by Dunnett's multiple comparison test against Collagen-I. A one-sample *t*-test was performed to assess differences in the anisotropic ratio compared to 1 (indicating no anisotropy). GraphPad Prism software was used. Results displayed as mean ± standard deviation and were considered statistically significant for ∗p < 0.05, ∗∗p < 0.01, ∗∗∗p < 0.001, ∗∗∗∗p < 0.0001.

## 3 Results

### 3.1 Fabrication of cardiac-specific and non-cardiac ECM scaffolds

**Fig. 1:**
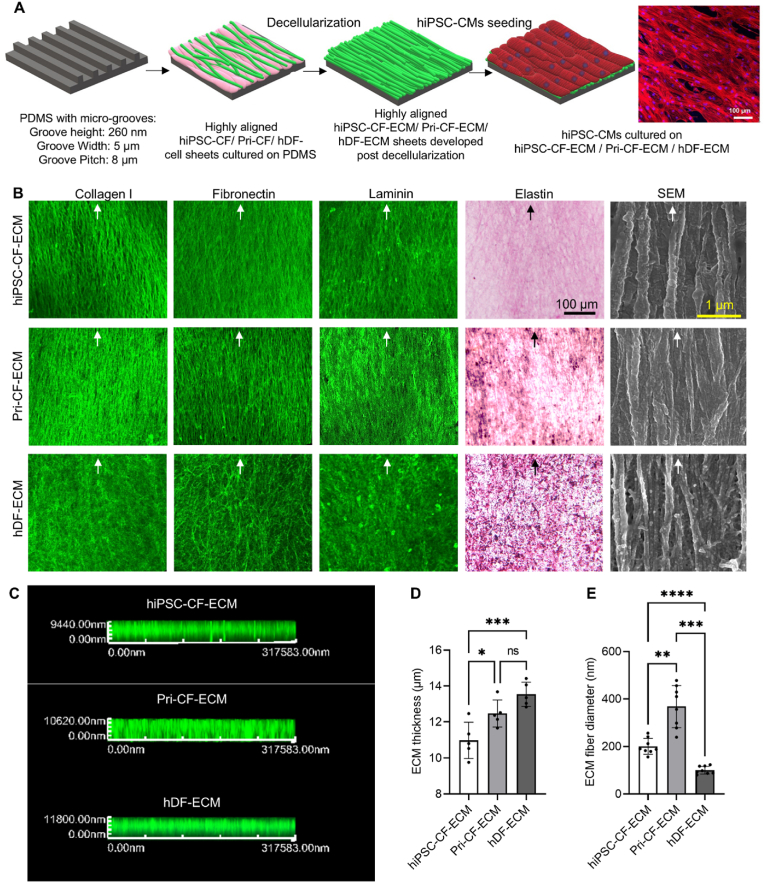
**Fabrication and characterization of ECM scaffolds.** (A) Schematic representation illustrating cardiac ECM (hiPSC-CF-ECM, Pri-CF-ECM) and non-cardiac ECM (hDF-ECM) fabrication for engineering hiPSC-CMs containing cardiac patches. (B) Immunofluorescence staining reveals the anisotropic and nanofibrous architecture of collagen-I, laminin and fibronectin within ECM sheets following surface topographical cues. Verhoeff Van Gieson staining revealed the organization of elastin fibers within ECM sheets. Anisotropic nano-scale ECM bundles organized in ECM sheets observed by FE-SEM images. (C, D) ECM thickness measured by Z-stacking in confocal imaging. (E) The thickness of nanofibrous ECM bundles quantified using FE-SEM micrographs. Results displayed as mean ± standard deviation and were considered statistically significant for ∗p < 0.05, ∗∗p < 0.01, ∗∗∗p < 0.001, ∗∗∗∗p < 0.0001.

**Fig. 2:**
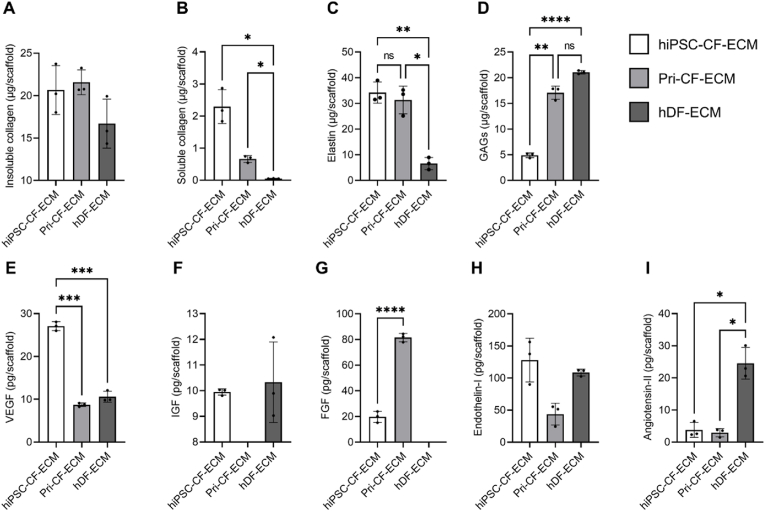
**Characterization of major ECM structural components and embedded growth factors.** Quantification of ECM-specific structural macromolecules including (A) insoluble collagen, (B) soluble collagen, (C) elastin, and (D) GAGs within ECM sheets post decellularization. Quantification of ECM embedded growth factors, including (E) VEGF, (F) IGF, (G) FGF, (H) endothelin I and (I) angiotensin II detected post decellularization. Results displayed as mean ± standard deviation and were considered statistically significant for ∗p < 0.05, ∗∗p < 0.01, ∗∗∗p < 0.001, ∗∗∗∗p < 0.0001.

Cardiac-specific ECMs (hiPSC-CF-ECM, Pri-CF-ECM) and a non-cardiac ECM (hDF-ECM) were prepared by growing highly aligned cell-sheets on micro-grated PDMS substrates for 2 weeks to allow for ECM secretion (Fig. 1A and S5). Subsequently, the cell sheets were decellularized to form highly aligned, nanofibrous ECM sheets, which were used as scaffolds to culture hiPSC-CMs, resulting in biomimetic cardiac tissues (Fig. 1A and S1). Immunofluorescence staining revealed a highly aligned and nanofibrous architecture of collagen-I, fibronectin, and laminin. Verhoeff Van Gieson staining showed the organization of elastin fibers in the ECM scaffolds. The fibers in the cardiac ECMs (hiPSC-CF-ECM and Pri-CF-ECM) displayed increased alignment compared to the non-cardiac hDF-ECM (Fig. 1B, white arrows). All three types of ECM had a similar thickness between 11 and 14 μm as observed by z-stacking in confocal imaging (Fig. 1C and D). Among the three, hDF-ECM and Pri-CF-ECM showed significantly higher thickness compared to hiPSC-CF-ECM, possibly due to their higher GAG content, which absorbs more water, leading to increased thickness (Fig. 2D). Sub-micron scale ECM fibrous bundles organized in the anisotropic orientation were observed by scanning electron microscopy (SEM). The average ECM fiber bundle diameters were highest for Pri-CF-ECM (396.62 ± 99.21 nm), followed by hiPSC-CF-ECM (196.62 ± 46.52 nm, *p* < 0.01) and hDF-ECM (104.59 ± 28.48 nm, *p* < 0.01; Fig. 1E).

### 3.2 Structural and compositional evaluation of ECM scaffolds

The structural and compositional characteristics of the three types of ECM scaffolds were analyzed. First, insoluble collagen, soluble collagen, elastin, and GAGs were quantified. hiPSC-CF-ECM and Pri-CF-ECM had non-significantly increased levels of insoluble collagen compared to hDF-ECM (Fig. 2A), while hiPSC-CF-ECM (2.29 ± 0.53 μg/scaffold) and Pri-CF-ECM (0.59 ± 0.24 μg/scaffold) showed significantly higher levels of soluble collagen than hDF-ECM (0.05 ± 0.04 μg/scaffold, *p* < 0.001). Additionally, the level of soluble collagen in hiPSC-CF-ECM was significantly higher than in Pri-CF-ECM (p < 0.01; Fig. 2B). Moreover, both hiPSC-CF-ECM (34.23 ± 4.11 μg/scaffold) and Pri-CF-ECM (31.33 ± 5.41 μg/scaffold) had significantly increased levels of elastin compared to hDF-ECM (6.61 ± 2.37 μg/scaffold, p < 0.01; Fig. 2C). Conversely, hDF-ECM had the highest level of GAGs (21.08 ± 0.33 μg/scaffold, *p* < 0.01). Compared to hiPSC-CF-ECM (4.90 ± 0.46 μg/scaffold), Pri-CF-ECM showed a significantly increased level of GAGs (17.08 ± 1.28 μg/scaffold; Fig. 2D).

Next, the ECM-embedded growth factors post-decellularization were quantified via ELISA. hiPSC-CF-ECM showed the highest concentration of the angiogenic growth factor VEGF (27.08 ± 1.04 pg/scaffold) compared to Pri-CF-ECM (8.72 ± 0.41 pg/scaffold) and hDF-ECM (10.59 ± 1.28 pg/scaffold, p < 0.01; Fig. 2E). On the other hand, IGF was not detected in Pri-CF-ECM and there was no significant difference between hiPSC-CF-ECM and hDF-ECM (Fig. 2F). Similarly, the levels of endothelin I in hiPSC-CF-ECM (128.02 ± 34.12 pg/scaffold, *p* < 0.05) and hDF-ECM (108.61 ± 5.33 pg/scaffold, *p* < 0.01) were significantly higher than Pri-CF-ECM (43.76 ± 16.92 pg/scaffold; Fig. 2H). In contrast, Pri-CF-ECM showed a significantly higher (*p* < 0.01) level of FGF (81.50 ± 3.32 pg/scaffold) than both hiPSC-CF-ECM (19.79 ± 4.36 pg/scaffold) and hDF-ECM (undetectable; Fig. 2G). The highest level (*p* < 0.01) of angiotensin II was detected in hDF-ECM (24.54 ± 4.94 pg/scaffold) compared to hiPSC-CF-ECM (3.83 ± 2.33 pg/scaffold) and Pri-CF-ECM (2.96 ± 1.29 pg/scaffold; Fig. 2I).

**Fig. 3:**
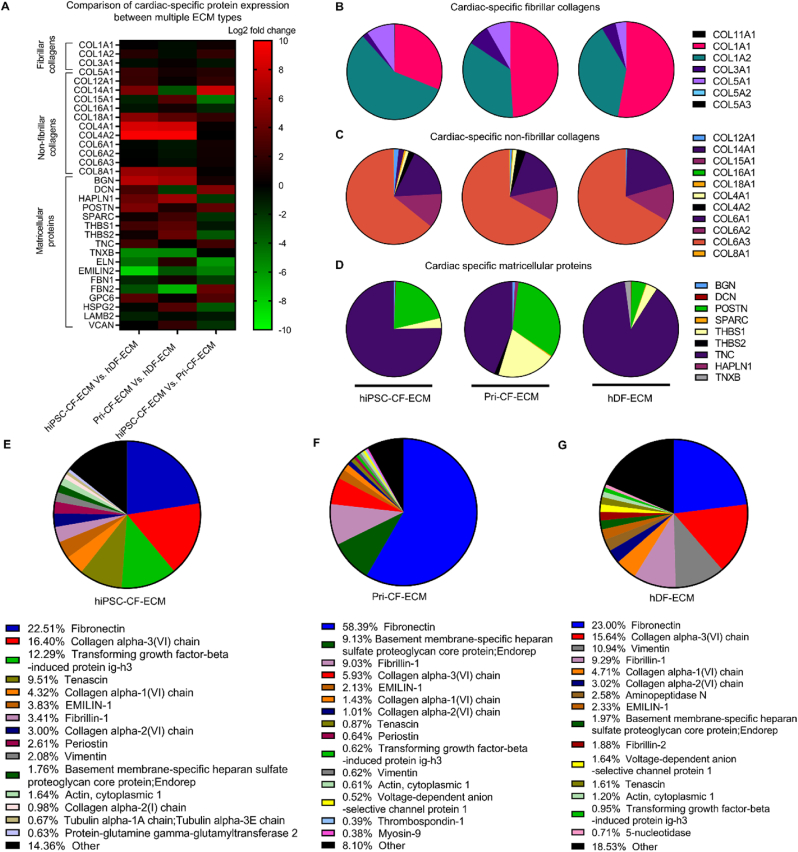
**Compositional evaluation of ECM scaffolds determined by mass spectrometry.** (A) Heat map comparing the fold difference in the expression of cardiac proteins between cardiac and non-cardiac ECM scaffolds. Pie graph of the relative abundance of (B) fibrillar collagens, (C) non-fibrillar collagens and (D) matricellular proteins, respectively. Pie graph of the most abundant 20 proteins in cardiac (E) hiPSC-CF-ECM, (F) Pri-CF-ECM, and non-cardiac (G) hDF-ECM, based on the LFQ intensities of each protein to indicate their abundance within the scaffold. LFQ intensities of the rest of the proteins identified were binned under other (black).

Then, an unbiased, complete compositional evaluation of the ECM scaffolds was performed via LC-MS analysis. Nearly 700 peptides were identified in each scaffold. Quantitated proteins were divided into two categories based on fold-change (FC): up-regulation (FC > 2) and down-regulation (FC < 0.5). Compared to Pri-CF-ECM, 86 proteins were upregulated (p < 0.05), and 290 proteins were downregulated (*p <* 0.05) in hiPSC-CF-ECM. When compared to non-cardiac hDF-ECM, 207 proteins were upregulated (*p* < 0.05), and 115 proteins were downregulated (p < 0.05) in hiPSC-CF-ECM (Supplementary data *sheet S1*). Label-free quantitation (LFQ) intensities were used as a proxy to determine protein abundance in each ECM type. Supplementary data sheet S1 contains a list of LFQ intensities indicating the abundance of all identified peptides in each ECM scaffold type. Fig. 3A and supplementary data sheet S2 indicate the log2 transformed fold changes for each protein for each pairwise comparison and the adjusted p-values calculated (Benjamini-Hochberg method) for the protein expression for each pairwise comparison. The ECM scaffolds were analyzed for the expression of cardiac fibrillar collagens, non-fibrillar collagens, and matricellular proteins. Both cardiac-ECMs, hiPSC-CF-ECM and Pri-CF-ECM, showed increased expression of fibrillar collagens (collagen type-I, III, V), non-fibrillar collagens (collagen type-IV, VIII, XII, XIV, XVIII), and matricellular proteins (BGN, DCN, HAPLN1, POSTN, THBS1, THBS2, TNC) compared to non-cardiac hDF-ECM (Fig. 3A). Moreover, hiPSC-CF-ECM and Pri-CF-ECM exhibited similar expression profiles of cardiac peptides (Fig. 3A). The relative abundance of the fibrillar and non-fibrillar collagens and matricellular proteins respectively revealed the presence of diverse fibrillar and non-fibrillar collagens that were abundant in hiPSC-CF-ECM but were absent in Pri-CF-ECM and hDF-ECM. (Fig. 3C and D, and Supplementary data sheet S2).

The 20 most abundant proteins for each ECM type were depicted separately in the pie graph, while the remaining proteins were grouped as “Other”. Therefore, the pie graphs in Fig. 3E–G showed the complete composition of hiPSC-CF-ECM (Fig. 3E), Pri-CF-ECM (Fig. 3F), and hDF-ECM (Fig. 3G). Across all three ECM types, fibronectin was the most abundant component, as indicated by the highest LFQ intensity. hiPSC-CF-ECM and hDF-ECM showed similar fibronectin levels (23 %; Fig. 3E–G), while Pri-CF-ECM showed an increased fibronectin content (58 %; Fig. 3F).

### 3.3 Evaluation of the structural and functional maturation of hiPSC-CMs on ECM scaffolds

**Fig. 4:**
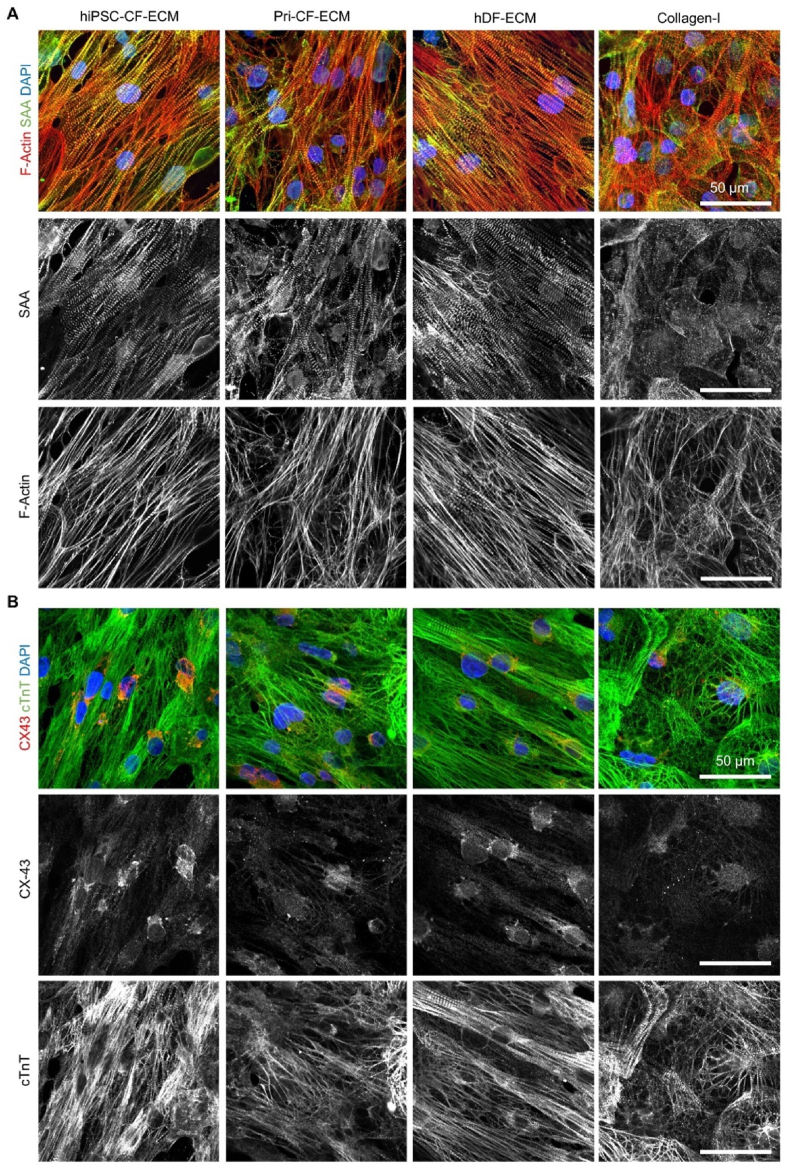
Organization of hiPSC-CMs on ECM *scaffolds. Anisotropic and matured organization of (A) sarcomeric* α*-actinin (SAA, green) and F-actin (red) and (B) cardiac troponin T (cTnT, green) and connexin 43 (CX43, red)) (Scale bar:* 50 μm*)*.

The potential of cardiac-specific ECMs to promote structural and functional maturation of hiPSC-CMs was evaluated via CM-specific structural and functional protein organization and gene expression analysis. hiPSC-CMs cultured on all three types of ECMs aligned with the fiber anisotropy and exhibited matured expression of structural proteins F-actin and sarcomeric α-actinin (SAA) oriented in the aligned direction. In contrast, hiPSC-CMs cultured on collagen-I-coated cover glass exhibited isotropic organization of F-actin and SAA (Fig. 4A and S1). Similarly, hiPSC-CMs cultured on the anisotropic ECM fibers showed matured organization of CM-specific functional proteins, *i.e.,* cardiac troponin T (cTnT) and connexin 43 (Cx43) (Fig. 4B).

**Fig. 5:**
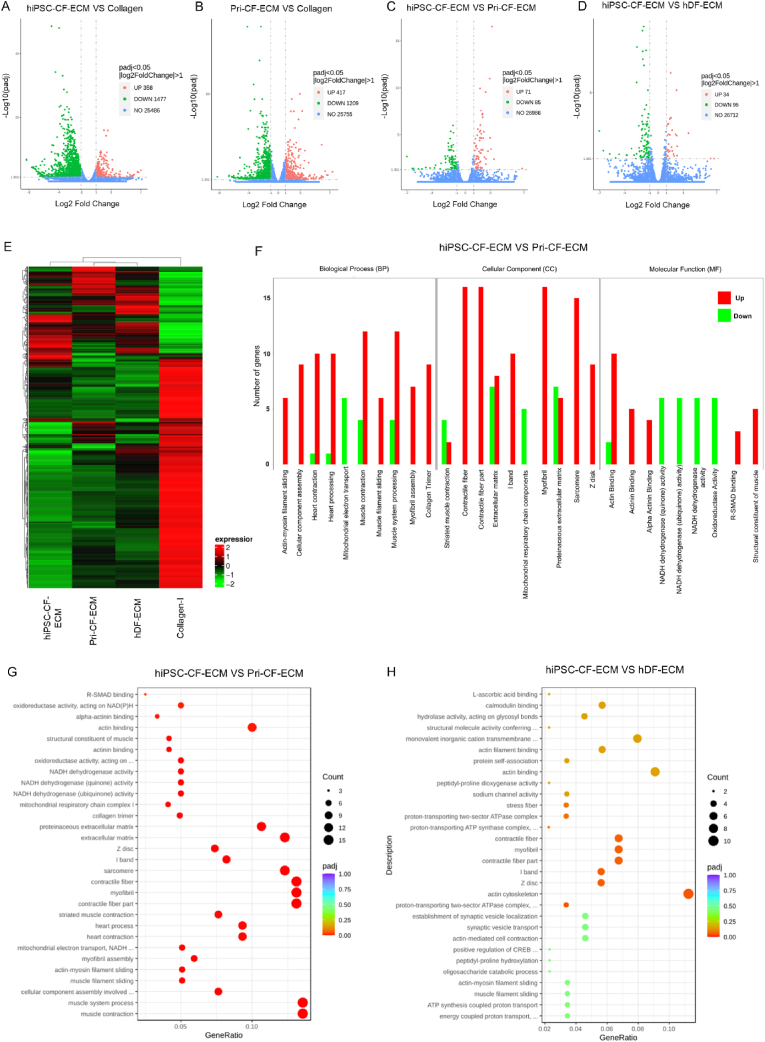
**Evaluating maturation of hiPSC-CMs cultured on ECM constructs via bulk RNA sequencing.** Differential gene volcano map indicating the number of up-regulated and down-regulated genes in hiPSC-CMs cultured on **(A)** hiPSC-CF-ECM Vs. collagen-I, **(B)** Pri-CF-ECM Vs. collagen-I, **(C)** hiPSC-CF-ECM Vs. Pri-CF-ECM, **(D)** hiPSC-CF-ECM Vs. hDF-ECM. **(E)** Hierarchical clustering of hiPSC-CMs cultured on hiPSC-CF-ECM, Pri-CF-ECM, hDF-ECM, and collagen-I based on mRNA expression obtained from RNA-seq. Heatmap indicating differential expression gene clustering from hiPSC-CMs cultured of various ECM constructs and collagen-I. **(F)** Gene ontology (GO) enrichment analysis reveals up/down regulation of cardiac-specific structural and functional genes that are activated in hiPSC-CMs cultured on hiPSC-CF-ECM versus Pri-CF-ECM. GO enrichment analysis scatter plot indicating expression of GO terms associated with cardiac-function in hiPSC-CMs cultured on **(G)** hiPSC-CF-ECM vs. Pri-CF-ECM, and **(H)** hiPSC-CF-ECM vs. hDF-ECM.

For gene expression analysis, whole transcriptome RNA-seq was conducted. After 7 days of culturing hiPSC-CMs on ECM scaffolds and collagen-I-coated cover glass, total RNA was extracted for bulk RNA-seq to identify differentially expressed genes (DEGs). Volcano plots were used to display the overall distribution of DEGs, with fold change in gene expression on the x-axis and statistical significance shown on the y-axis (Fig. 5A–D). Upregulated genes were marked by red dots and downregulated genes were indicated by green dots. hiPSC-CMs cultured on cardiac-specific ECM scaffolds showed a significant (P-adj <0.05, log2Fold-change >1) change in their gene expression profile compared to hiPSC-CMs cultured on collagen-coated-cover glass (Fig. 5A and B). In contrast, no significant difference was noted in the gene expression profile of hiPSC-CMs cultured on ECM scaffolds. In addition, hiPSC-CMs cultured on hiPSC-CF-ECM showed 71 upregulated genes and 85 downregulated genes compared to Pri-CF-ECM (Fig. 5C), while hiPSC-CMs cultured on hiPSC-CF-ECM showed upregulation of 34 genes and downregulation of 96 genes compared to the hDF-ECM (Fig. 5D) (P-adj <0.05, log2Fold-change >1).

Next, cluster analysis was performed to group genes with similar expression patterns. Mainstream hierarchical clustering was used to cluster the fragments per kilobase of transcript per million mapped reads (FPKM) values of genes and homogenize the row (Z-score). Genes or samples with similar expression patterns in the heat map were gathered, and the overall results were clustered using the log2 (FPKM+1) value. The color ranging from red to green indicates log2(FPKM+1) values from large to small (Fig. 5E). The heat map revealed a significant change in the gene expression in hiPSC-CMs cultured on cardiac and non-cardiac ECM scaffolds, and collagen-coated cover-glass (Fig. 5E and S2). DEG clustering revealed significant changes in the expression profile of hiPSC-CMs cultured on collagen-I compared to those cultured on the ECM-scaffolds. Notably, the gene expression profile of hiPSC-CMs cultured on Pri-CF-ECM more closely resembled that on hDF-ECM than that on hiPSC-CF-ECM (Fig. 5E and S2).

Gene Ontology (GO) analysis was then performed, selecting the 30 most significant GO terms and categorizing them into biological processes, cell components, and molecular functions. This analysis revealed the cardiac-specific structural and functional genes activated in hiPSC-CMs cultured on the ECM scaffolds. Among the cardiac ECM scaffolds, hiPSC-CMs cultured on hiPSC-CF-ECM showed increased expressions of CM-specific genes associated with biological processes (*i.e.*, actin-myosin filament sliding, heart and muscle contraction, heart processing, myofibril assembly, and collagen trimer formation), molecular functions (*i.e.*, actin/actinin/α-actinin binding, R-SMAD binding, structural components of the muscles), and cellular components *(i.e.*, contractile fiber components, I band, Z-disk, sarcomere, myofibril) compared to on Pri-CF-ECM (Fig. 5F). Moreover, compared to on the non-cardiac hDF-ECM, hiPSC-CMs cultured on hiPSC-CF-ECM showed increased expressions of CM-specific genes associated with biological process (*i.e.*, actin-myosin filament sliding, muscle filament sliding, actin mediated cell-contraction), molecular functions (*i.e.*, actin binding, calmodulin binding, protein self-association) and cellular components (*i.e.*, contractile fiber, I band, Z disk, myofibril) (Fig. S2B). GO enrichment analysis histograms showed that hiPSC-CMs cultured on hiPSC-CF-ECM showed increased expression of GO terms associated with myofilaments, muscle-myosin complex, muscle α-actinin binding, Z disk, I-band, actin-mediated cell contraction, muscle contraction, heart contraction, heart processing in all three comparison groups including hiPSC-CF-ECM vs. Collagen-I, hiPSC-CF-ECM vs. hDF-ECM and hiPSC-CF-ECM vs. Pri-CF-ECM (Fig. S3A–C). However, the top 30 GO terms observed in comparison groups excluding hiPSC-CF-ECM such as Pri-CF-ECM vs. Collagen-I, Pri-CF-ECM vs. hDF-ECM, and hDF-ECM vs. Collagen-I were not cardiac-function-specific (Fig. S3D–F). These data further reinforced the significance of hiPSC-CF-ECM in promoting cardiac-specific functional maturation of hiPSC-CMs.

Furthermore, the gene ratio was calculated as the ratio between the number of differentially expressed genes in each GO term and that of all differentially expressed genes in the GO database. Gene ratio analysis of GO terms associated with cardiac function revealed that hiPSC-CMs cultured on hiPSC-CF-ECM showed significantly increased levels of genes associated with actin binding, Z-disk, and I-band formation, sarcomere structure, contractile fiber and myofibril formation, actin cytoskeleton, striated muscle contraction, heart contraction and heart processing compared to on Pri-CF-ECM and hDF-ECM (Fig. 5G and H). A greater difference in the expression of the GO terms was observed in hiPSC-CF-ECM vs. Pri-CF-ECM (Fig. 5G) compared to hiPSC-CF-ECM vs. hDF-ECM (Fig. 5H). However, the top 30 GO terms observed in comparison groups excluding hiPSC-CF-ECM, such as Pri-CF-ECM vs. Collagen-I, Pri-CF-ECM vs. hDF-ECM, and hDF-ECM vs. Collagen-I, were not cardiac-function-specific and mainly associated with ECM component, chemotaxis, cell migration, regulation of system process, GAG binding, hormone activity, integrin binding, collagen trimer, positive regulation of cell development, and cyclic nucleotide metabolic process (Fig. S4). Overall, the hiPSC-CF-ECM group exhibited the highest expression of genes associated with cardiac function and processing, indicating the significance of hiPSC-CF-ECM in improving the functional maturation of hiPSC-CMs (Fig. 5).

### 3.4 Contractile properties of hiPSC-CMs on different ECM

**Fig. 6:**
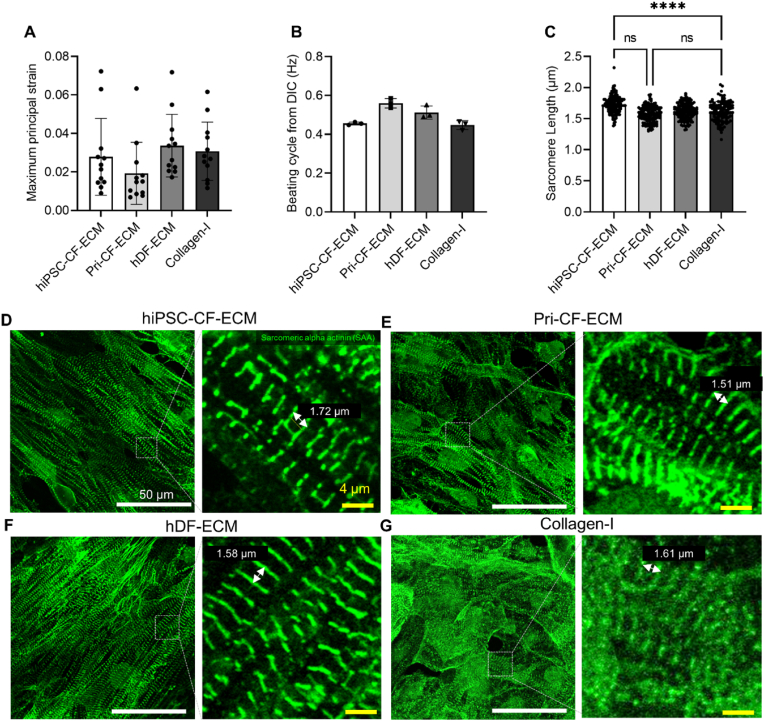
Evaluating contractile properties of hiPSC-CMs. Digital image correction (DIC)-based analysis. **(*A)****maximum principal strain variation and****(B)****beating cycle of hiPSC-CMs cultured on ECM scaffolds and their comparison with collagen-I-coated cover glass measured* via *DIC.****(C)****immunofluorescence staining-based sarcomere length measurement. hiPSC-CMs cultured on hiPSC-CF- ECM****(D)****, Pri-CF-ECM****(E)****, hDF-ECM****(F)****, and****(G)****collagen-I-coated cover glass was stained with sarcomeric* α−*actinin (SAA) to measure the distance between the two SAA signals. Results displayed as mean ± standard deviation and were considered statistically significant for ∗p < 0.05, ∗∗p < 0.01, ∗∗∗p < 0.001, ∗∗∗∗p < 0.0001.*

The contractile function of hiPSC-CMs was evaluated using videos of the beating CMs after 7 days of culture on ECM scaffolds or collagen-I-coated cover glass. The captured videos were analyzed using the DIC method to determine the correlation between reference and deformed images obtained from the beating videos. The displacement vectors of each subset were calculated, and the maximum principal strain values in the CMs were computed from the displacement vector fields. DIC-based analysis revealed no significant difference in maximum principal strains during beating cycles (Fig. 6A) and no significant difference in beating rates as measured by the number of periodic maximum principal strain variation cycles during a fixed time among hiPSC-CF-ECM, Pri-CF-ECM, hDF-ECM, and collagen-I groups (Fig. 6B, Supplementary videos). Sarcomere length was measured via two consecutive signals obtained from immunofluorescence staining of SAA (Fig. 6C and D). The hiPSC-CF-ECM group showed significantly increased sarcomere length compared to the other experimental groups (p < 0.001), which is consistent with a greater degree of CM maturity (Fig. 6C).

### 3.5 Electrophysiological properties of hiPSC-CMs on different ECM

**Fig. 7:**
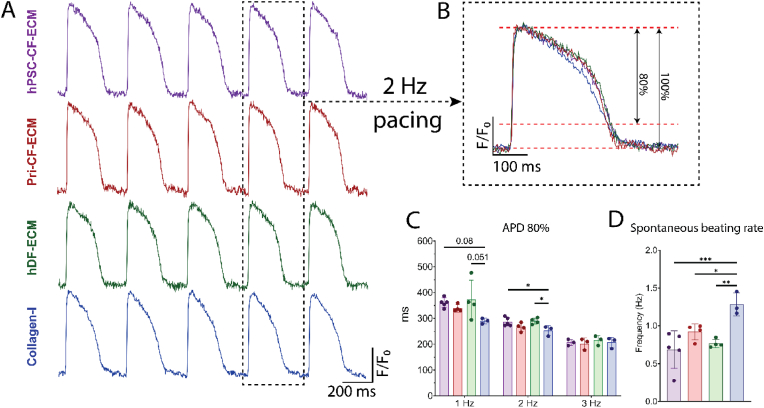
**Optical action potentials recorded from hiPSC-CM monolayers cultured on different ECM scaffolds. (A**–**B)** Representative action potentials shown are shown for monolayers seeded on PDMS with hiPSC-CF-ECM, Pri-CF-ECM, hDF-ECM and Collagen-I (glass coverslips) coatings with electrical pacing 2 Hz. **(C)** Summarized data for action potential duration at 80 % repolarization. **(D)** Spontaneous beating rate. hiPSC-CF-ECM n = 5, Pri-CF-ECM n = 4, hDF-ECM n = 4, Collagen-I n = 3. *P* values were determined by one-way ANOVA with multiple comparisons to the collagen-I group. Data represented as mean ± SD. ∗p < 0.05, ∗∗p < 0.01, ∗∗∗p < 0.005.

Cells were loaded with a fluorescent voltage-sensitive dye (FluoVolt™) to evaluate the electrophysiology of the hiPSC-CM monolayers cultured on the different ECMs. All cultures exhibited spontaneous action potentials, although the rate was faster for hiPSC-CMs cultured on collagen-I compared to the other conditions (Fig. 7D). The cultures were paced at different frequencies. Action potential duration at 80 % of repolarization (APD80) showed a progressive decrease at increasing stimulation frequency for each condition, consistent with rate-adaptation typical of cardiac muscle. The hiPSC-CMs cultured on collagen-I had a shorter APD80 than hiPSC-CF-ECM and hDF-ECM at 2 Hz, with a trend at 1 Hz, but there were no significant differences among groups at 3 Hz stimulation.

**Fig. 8:**
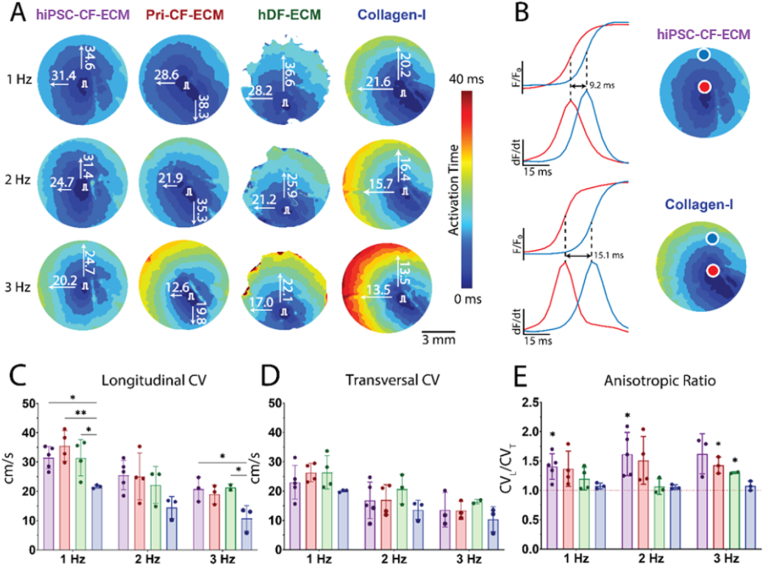
**Influence of the nanofibrous ECM scaffolds on the electrical conduction properties of hiPSC-CM monolayers. (A**–**B)** Representative voltage activation maps are shown for monolayers seeded on PDMS with hiPSC-CF-ECM, Pri-CF-ECM, hDF-ECM, and Collagen-I (glass coverslips) coatings. Activation maps were reconstructed during electrical pacing (1, 2, 3 Hz). Arrows show the directions of CV measurements (in cm/s), including longitudinal (aligned with PDMS chips pattern – CV_L_) and transversal (CV_T_). All the maps are plotted within the same time scale for comparison. **(C**–**E)** Summarized data for CV_L_, CV_T,_ and anisotropy ratio (CV_L_/CV_T_) measured in all groups at different frequencies. hiPSC-CF-ECM n = 3∼5, Pri-CF-ECM n = 3∼4, hDF-ECM n = 2∼4, Collagen-I n = 3. *P* values were determined by one-way ANOVA with multiple comparisons to Collagen-I (C–D) and one sample *t*-test (E). Data represented as mean ± SD. ∗p < 0.05, ∗∗p < 0.01.

Optical mapping of the fluorescent voltage signals for electrical impulse conduction through the monolayers assayed for anisotropic conduction, an important physiological characteristic of cardiac tissue [46]. The hiPSC-CM monolayers cultured on patterned PDMS chips exhibited significantly faster CV_L_ compared to those on glass coverslips coated with collagen-I when paced with 1 Hz: 31.5 ± 3.8 cm/s for hiPSC-CF-ECM (p = 0.03), 35.5 ± 5.2 cm/s for Pri-CF-ECM (p = 0.005), 31.4 ± 6.2 cm/s for hDF-ECM (p = 0.039), and 21.6 ± 0.6 cm/s for collagen-I (Fig. 8A–C). At 2 Hz and 3Hz, CV_L_ was the slowest in the collagen-I group, but the difference did not reach significance for all groups. In contrast, no significant differences in CV_T_ were detected among all samples across all stimulation frequencies. All conditions showed a decrease in CV as the stimulation frequency increased.

To quantify anisotropy levels, a one-sample *t*-test was conducted with a no-anisotropy assumption set at a CV_L_/CV_T_ anisotropy ratio of 1 (Fig. 8E). At 1 Hz stimulation, the ratio was significantly identified in hiPSC-CF-ECM (1.41 ± 0.2, p = 0.014) and exhibited a trend in Pri-CF-ECM (1.37 ± 0.3, p = 0.306). In hDF-ECM, AR was 1.20 ± 0.02 (p = 0.128), while in collagen-I, it was 1.08 ± 0.05 (p = 0.119). Similar trends were observed at 2 and 3 Hz stimulation. Overall, these data are consistent with the structural cues from the patterned ECM enabling anisotropic conduction in the monolayer hiPSC-CM cultures.

### 3.6 hiPSC-CF-ECM rescues MI in rat model

**Fig. 9:**
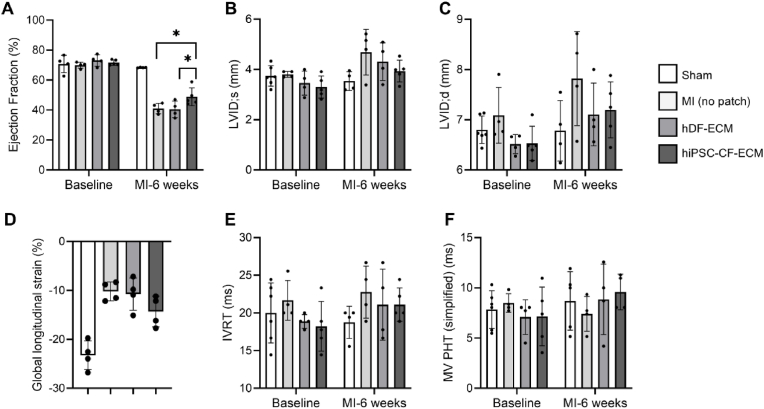
**Echocardiographic assessment of cardiac function in a rat myocardial infarction (MI) model following hiPSC-CF-ECM implantation.** (A) Ejection fraction (EF) quantified from B-mode imaging in PSLAX; (B) Left ventricular internal diameter during systole (LVID; s) and (C) Left ventricular internal diameter during diastole (LVID; d), both assessed via PSLAX M-mode; (D) Global longitudinal strain (GLS) calculated using speckle-tracking echocardiography; (E) Isovolumic relaxation time (IVRT) and (F) mitral valve pressure half-time (PHT), both measured from pulsed-wave Doppler spectra. Data are presented as mean ± standard deviation; statistical significance (∗p < 0.05) was determined by one-way ANOVA with post hoc multiple comparisons.

Cardiac impairment is characterized by dysfunction of the left ventricle, maladaptive remodeling, and the development of heart failure. To evaluate the *in vivo* efficacy of our ECM, we transplanted hiPSC-CF-ECM and hDF-ECM onto the MI region in SD rats. The sheets were sutured to the heart and remained intact on the beating heart (Fig. S6). Rats treated with hiPSC-CF-ECM exhibited improved myocardium contractility, as shown by the significantly improved ejection fraction compared to those treated with non-cardiac specific hDF-ECM (Fig. 9A).

Left ventricular internal dimension in systole (LVID; s) and diastole (LVID; d) are key measurements indicating the internal diameter of the left ventricle, representing ventricular remodeling in response to injury (Fig. 9B and C). Increased LVID; s and LVID; d values indicate dilation and poor heart contraction. Compared to the MI (no patch) and hDF-ECM groups, lowered LVID; s and LVID; d values in the hiPSC-CF-ECM group indicate reduction in dilation and improved contraction (Fig. 9B and C). Global longitudinal strain (GLS) is a direct measure of myocardial deformation, which is expressed as a negative percentage indicating shortening of heart muscle fibers in the longitudinal direction during contraction. Consistent with the ejection fraction values, the hiPSC-CF-ECM group showed enhanced GLS values compared to the MI (no patch) and hDF-ECM groups, indicating improved myocardial function (Fig. 9D). Isovolumic relaxation time (IVRT) measures the time between closing of the aortic valve and opening of the mitral valve during diastole. A prolonged IVRT represents diastolic dysfunction indicating heart's failure to relax properly. Compared to the MI (no patch) group, both hDF-ECM and hiPSC-CF-ECM groups showed a reduction in IVRT (Fig. 9E). Similarly, mitral valve pressure half-time (MV PHT) reflects the blood flow through the mitral valve and relaxation of the left ventricle. Shortened MV PHT values in the MI (no patch) group compared to the sham and hiPSC-CF-ECM groups may indicate mitral regurgitation, resulting in blood flow retrograding into the left atrium (Fig. 9F). Overall, echocardiography-based evaluation of our engineered cardiac patch in a rat MI model indicates that hiPSC-CF-ECM potentially contributes toward positive cardiac remodeling.

## 4 Discussion

We have developed an hiPSC-CF-ECM scaffold with a bioactive, anisotropic structure enriched in growth factors and cardiac proteins. This scaffold offers mechanical stability, complex composition, and reduced batch-to-batch variation, making it a universal, patient-personalizable option. This study investigates hiPSC-CF-ECM's role in enhancing hiPSC-CM maturation and function in vitro, explores the mechanisms of cardiac protein influence, and assesses its therapeutic efficacy in restoring cardiac function post-MI in vivo, using hDF-ECM as controls to highlight the benefits of cardiac specificity.

Quantification of structural components revealed that hiPSC-CF-ECM contained significantly higher or comparable levels of soluble collagen, insoluble collagen, and elastin compared to Pri-CF-ECM and hDF-ECM (Fig. 2A–C). These components contribute to the mechanical strength of the engineered ECM scaffolds, consistent with our previous findings [36]. The decellularization process preserved ECM-embedded growth factors, including VEGF, IGF, FGF, endothelin-I, and angiotensin-II, in the engineered cardiac ECMs (Fig. 2E–I). These growth factors are essential for supporting CM growth and maturation, enhancing the bioactivity of our ECM platform. This aligns with past studies, where VEGF and FGF secretion was reported from human CFs [47] and angiotensin-II synthesis from canine CFs [48]. Among these growth factors, IGF is critical for stimulating heart contractility and tissue-remodeling, improving heart function post-MI [49]. Multiple cardiac sub-populations including CMs and ECs express VEGF receptors (VEGFR-1 and VEGFR-2), which, upon VEGF binding, trigger morphogenesis, vasculogenesis, contractility, and healing post-MI [50]. Endothelin-1, a potent vasoconstrictor with inotropic and mitogenic effects, regulates salt-water homeostasis, vascular tone, and blood pressure while promoting CM hypertrophic remodeling [51,52]. Our results showed that the levels of VEGF, IGF, and endothelin-I in hiPSC-CF-ECM were significantly higher or comparable to those in Pri-CF-ECM and hDF-ECM (Fig. 2E, F, H). Additionally, FGFs are crucial for cardiac homeostasis via heart morphology, physiology, CM proliferation, myocardial excitability, angiogenesis, and energy balance regulation [53]. Angiotensin-II regulates contractility, cell coupling, and impulse propagation, and is involved in cardiac remodeling, growth, and apoptosis [54]. Lower levels of angiotensin-II in both cardiac ECMs compared to hDF-ECM may contribute to reduced adverse cardiac remodeling post-MI (Fig. 2I) [55].

Fibrillar collagens, particularly collagen I and V, constitute about 70 % of adult human cardiac ECM [56], with basement membrane proteins, including collagen IV, agrin, laminin, perlecan, and nidogen, comprising around 20 % of cardiac ECM. Fibrous glycoproteins and proteoglycans form 4 % of adult cardiac ECM, while matricellular proteins, including collagen VI, lumican, prolargin, periostin, thrombospondin-2, fibronectin, emilin, dermatopontin, and fibulin, account for 3 % [57,58]. LC-MS analysis confirmed the presence of native cardiac fibrillar, non-fibrillar, and matricellular proteins in the engineered CF-ECM scaffolds (Fig. 3). As predicted, hiPSC-CF-ECM and Pri-CF-ECM showed the presence of multiple cardiac proteins, especially fibrillar and non-fibrillar collagens, compared with hDF-ECM (Fig. 3E and F). While Pri-CF-ECM exhibited higher levels of fibrillar (COL3) and non-fibrillar (COL15, COL16) collagens than hiPSC-CF-ECM, the majority of the fibrillar collagens (COL1, COL5) and non-fibrillar collagens (COL12, COL14, COL18) were highly expressed in hiPSC-CF-ECM (Fig. 3 and Supplementary Data Sheet S2). Collagens such as COL1 [59,60], COL3 and COL5 [61], and COL14 [62] were reported to enhance CM maturation. COL1 achieves this via the stimulation of collagen integrins α2 and β1 [60], while COL14 may do so indirectly through promoting proper collagen fibrillar network organization and remodeling [62]. Compared to hiPSC-CF-ECM, hDF-ECM has similar levels of COL1 but lower levels of COL5 and COL14. The upregulation of structural proteins in hiPSC-CF-ECM may be attributed to elevated transforming growth factor β (TGF-β) signaling, as indicated by the presence of TGF-β-induced protein ig-h3 [36]. The lower collagen content and possibly lower stiffness in hiPSC-CF-ECM could promote more CM dedifferentiation and proliferation [63].

hiPSC-CF-ECM also exhibited higher levels of certain pro-regenerative matricellular proteins [64], such as periostin (POSTN) and tenascin C (TNC), but lower levels of others, including versican (VCAN), fibronectin (FN1), elastin microfibril interfacer 2 (EMILIN2), heparan sulfate proteoglycan 2 (HSPG2), and hyaluronan and proteoglycan link protein 1 (HAPLN1), compared to Pri-CF-ECM. Compared to hDF-ECM, hiPSC-CF-ECM has higher POSTN, TNC, and HAPLN1, but lower EMILIN2. POSTN is deposited by a subset of CFs from postnatal days 1–11 and promotes cardiac nerve development and CM maturation [65]. It also induces proliferation in differentiated CMs via α5, β1, β3, and β5 integrins [65,66]. TNC promotes CM migration and invasion at the infarct-myocardium boundaries in zebrafish [67]. VCAN is upregulated following neonatal myocardial injury, with intramyocardial injection of this ECM protein enhancing CM proliferation and improving cardiac function via integrin β1 activation [68]. Similarly, epicardial FN1 promotes CM proliferation via integrin β1 activation [69] and supports CM maturation in 3D-engineered heart tissues [70]. In a zebrafish model, EMILIN2, expressed by coronary endothelial cells and epicardium-derived cells, induces the expression of the chemokine gene cxcl8a, which promotes coronary revascularization during cardiac regeneration [71]. HSPGs promote infarct healing and prevent post-MI heart failure by providing binding sites for growth factors such as VEGF and cytokines and retaining water [72]. HAPLN1, secreted by epicardial cells, retains water and is essential for CM proliferation during cardiac regeneration in zebrafish [73].

Overall, the unique protein composition of hiPSC-CF-ECM may contribute to its potential for supporting functional recovery post-MI by balancing cardioprotective and structural elements differently than Pri-CF-ECM, as evident by bulk RNA-seq analysis (Fig. 5). hiPSC-CF-ECM and Pri-CF-ECM exhibit matrisome profiles closely resembling those of fetal cardiac ECM. In embryonic mice, CFs show higher RNA expression of POSTN, TNC, FN1, HAPLN1, COL5A2, COL3A1, and COL12A1 than their adult counterparts [69]. Similarly, fetal human CFs are enriched in TNC, COL6A1, COL6A2, COL6A3, and fibrillin-2 , while adult human CFs express higher levels of TGF-β [74]. Williams et al. [23] and Ozcebe et al. [29] used LC-MS to analyze the composition of decellularized fetal and adult mouse cardiac ECM and adult human cardiac ECM, respectively, and showed that while adult cardiac ECM promotes CM maturation, only young developmental age cardiac ECM promotes CM proliferation. Notably, all of our ECM scaffolds contained lower levels of periostin than fetal mouse ECM (Supplementary Table 2), likely due to the absence of cardiac endothelial cells that produce periostin [75]. Additionally, key cardiac-regenerative ECM proteins agrin and reelin (secreted by endothelial cells) and osteopontin (secreted by immune cells) were not detected [75]. This limitation could be addressed by co-culturing hiPSC-derived endothelial cells in the cell sheet and decellularizing the resulting capillaries to obtain their basement membrane proteins [76]. In addition, since hiPSC-CF-ECM is derived from cells cultured under sterile conditions, it has a lower pathogen transmission risk than donor-derived tissues. Deriving ECM from cells also allows greater control over donor variability, as one iPSC line has the capacity to produce much more ECM than a single donor organ could provide. There are also various in vitro strategies to reduce iPSC variation that would not be applicable to donor tissues [77]. Unfortunately, since cardiac-regenerative ECM proteins were identified through large-scale screens and tested individually, their potential synergistic effects in combinations remain unknown [75]. Therefore, future *in vivo* studies are needed to evaluate the impact of the compositional differences between hiPSC-CF-ECM and Pri-CF-ECM.

All three ECM scaffolds effectively aligned the hiPSC-CMs along the anisotropic ECM fibers. After 7 days of culture on engineered ECM scaffolds, hiPSC-CMs displayed more mature expression of myofilament proteins (F-actin and SAA; Fig. 4A, and cTnT Fig. 4B). RNA-seq analysis revealed that the hiPSC-CMs cultured on all three ECM scaffolds had a distinct expression profile compared to those cultured on collagen-I coated cover glass (Fig. 5E and S2A). Among the three ECM groups, hiPSC-CMs cultured on hiPSC-CF-ECM showed the most evidence of maturation, with upregulated expression of mitochondrial/oxidative metabolism genes and increased expression of myofilament and sarcomeric genes, compared to the Pri-CF-ECM and hDF-ECM groups (Fig. 5 and S2-4). Furthermore, hiPSC-CMs cultured on hiPSC-CF-ECM showed the longest sarcomere length consistent with the greatest maturity (Fig. 6C).

A matured sarcomere structure with uniform contractile beating in the anisotropic direction was clearly observed across all groups, with the effect being more prominent in the hiPSC-CF-ECM and hDF-ECM groups compared to the Pri-CF-ECM group (Fig. 4A and Supplementary Videos). Among the experimental groups, Pri-CF-ECM exhibited the shortest sarcomere length (Fig. 6C, D-G). The quantification of periodic variation of contractile beating in sarcomere revealed no significant differences in maximum principal strain or beating period. In the current study, only in-plane deformation and strain were evaluated, without considering out-of-plane distributions. Moreover, hiPSC-CM monolayers cultured on cardiac and non-cardiac ECM sheets exhibited enhanced CV_L_ compared to those grown on collagen I-coated glass coverslips. This improvement is attributed to the cell alignment along the ECM pattern, which supports conduction in the longitudinal direction. The slower spontaneous rate of contraction observed for monolayers on ECM sheets relative to collagen in optical mapping experiments is consistent with enhanced maturation and an associated decrease in automaticity of the hiPSC-CMs [78,79]; differences in maturation may also contribute to the shorter APD80 observed on collagen compared to the ECM. Additionally, the lower stiffness of PDMS compared to that of glass potentially favors the maturation and enhanced function of the hiPSC-CMs, as cells can sense the stiffnesses of the underlying substrate through the ECM or collagen at the thicknesses that we used [80,81]. Anisotropic conduction was evident in hiPSC-CM monolayers seeded on hiPSC-CF-ECM and Pri-CF-ECM, but not on hDF-ECM or collagen-I on glass coverslips (Fig. 8). These findings support the hypothesis that hiPSC-CMs exhibit anisotropic conduction when cultured on cardiac-specific ECM scaffolds with anisotropic alignment.

Maturation occurs on multiple levels during cardiac development in response to a myriad of signaling processes, and key aspects of this process are observed in vitro. In the present study, the greatest evidence for hiPSC-CM maturation on hiPSC-CF-ECM is the significant increase in sarcomere length and the greatest increase in expression of genes associated with oxidative metabolism. A reduction in spontaneous rate is another indicator of maturation based on changes in ionic currents associated with automaticity during development, and this was observed for hiPSC-CMson all ECMs relative to on collagen. However, strain measurements to assess contractility did not detect differences between groups with increased strain anticipated to reflect advanced maturity. The variable metrics of maturation among the groups reflect the complexity of maturation, which is consistent with prior studies that have demonstrated that distinct interventions differentially impact maturation properties of hiPSC-CMs [82,83]. How the different signaling from the ECMs impact different aspects of maturation is an important question for future studies including efforts to advance maturation even further towards adult cardiac tissue. The combination of advanced maturation and anisotropic organization makes the hiPSC-CM on hiPSC-CF-ECM the most physiologically relevant platform studied and well positioned for future studies investigating cardiac biology, disease, or drug development. Likewise, having the most mature iPSC-CM preparation with rapid anisotropic conduction is optimal for tissue engineering applications for cardiac repair.

Finally, hiPSC-CF-ECM demonstrated promising therapeutic potential in rat MI model. The cardiac-regenerative protein composition and the highly organized nanofibrous structure allowed hiPSC-CF-ECM to improve systolic function in rat hearts six weeks after MI. (Fig. 9), showcasing hiPSC-CF-ECM's therapeutic effect on cardiac remodeling and regeneration as a transformative cardiac therapy. These results are in agreement with a similar study by Shah et al. [84], where they implanted 600 μm thick decellularized porcine myocardium slices on infarcted rat myocardium and reported an ejection fraction of 60 % after 4 weeks. A limitation of the present study is the lack of in-depth investigation into the underlying mechanisms of hiPSC-CF-ECM's therapeutic effects and its immunological safety assessment in the rat MI model, as this was beyond the scope of the proof-of-concept animal experiment design. While the overall impact of hiPSC-CF-ECM on cardiac function was demonstrated, future studies are planned to comprehensively assess the effects of all ECMs on post-MI cardiac remodeling through biochemical, molecular, and histological analyses.

So far, ECM-based cardiac patches have primarily used either reconstituted individual ECM components or ECM derived from human or animal tissues [85]. Compared to decellularized donor tissue cardiac patches, our lab-grown hiPSC-CF-ECM offers lower batch-to-batch variation, reduced pathogen transfer risks, and higher customizability. Compared to 3D-printed cardiac patches, hiPSC-CF-ECM provides higher growth factor content, a more biomimetic chemical composition, and greater fiber structure resolution [9,86,87]. Our strategy presents an innovative "bottom-up" approach for engineering completely biological, highly anisotropic, and cardiac-specific ECM scaffolds to advance functional cardiac tissues.

## 5 Conclusions

A completely biological and anisotropic cardiac-specific ECM scaffold was developed by decellularizing cardiac fibroblast sheets. The hiPSC-CF-ECM scaffolds showed the highest expression levels of native cardiac ECM-specific fibrillar collagens, non-fibrillar collagens, and matricellular proteins compared to other scaffolds. hiPSC-CM cultured on these engineered ECM scaffolds exhibited a matured organization of CM-specific structural and functional proteins. Bulk-RNA-seq, optical mapping, and DIC-based strain measurement analysis indicated that hiPSC-CF-ECM promoted functional maturation of hiPSC-CMs by stimulating expression of cardiac-specific structural and functional genes, possibly by restoring the critical cell-ECM crosstalk. This novel bottom-up engineering approach to fabricate a cardiac-specific ECM scaffold represents a promising platform to engineer functional cardiac tissues for regenerative medicine applications.

## CRediT authorship contribution statement

**Dhavan Sharma:** Writing – original draft, Methodology, Investigation, Formal analysis, Data curation. **Wenkai Jia:** Methodology, Investigation, Formal analysis, Data curation. **Alvis Chiu:** Methodology, Investigation, Formal analysis, Data curation. **Hee Jae Jang:** Investigation, Formal analysis, Data curation. **Vladislav Leonov:** Methodology, Investigation, Formal analysis, Data curation. **Zhishi Chen:** Methodology, Investigation, Formal analysis, Data curation. **Brandon Zhao:** Methodology, Investigation, Data curation. **Weijia Luo:** Methodology, Investigation, Formal analysis, Data curation. **Hutomo Tanoto:** Methodology, Investigation, Formal analysis, Data curation. **Jianhua Zhang:** Methodology, Formal analysis, Data curation. **Alexey V. Glukhov:** Validation, Supervision, Methodology. **Yong Yang:** Writing – review & editing, Validation, Supervision, Methodology. **Yuxiao Zhou:** Writing – review & editing, Supervision, Methodology, Formal analysis. **Jiang Chang:** Writing – review & editing, Validation, Supervision, Methodology, Funding acquisition, Formal analysis, Conceptualization. **Timothy J. Kamp:** Writing – review & editing, Validation, Supervision, Resources, Project administration, Methodology, Funding acquisition, Conceptualization. **Feng Zhao:** Writing – review & editing, Writing – original draft, Supervision, Resources, Project administration, Methodology, Funding acquisition, Conceptualization.

## Ethics approval and consent to participate

All animal experiments received approval from the Institutional Animal Care and Use Committee of the Texas A&M University Health Science Center-Houston, under protocol number IACUC 2023-0043-H.

## Declaration of competing interest

Feng Zhao is an editorial board member for Bioactive Materials and was not involved in the editorial review or the decision to publish this article. All authors declare that there are no competing interests.

## Appendix A Supplementary data

Multimedia component 3 Multimedia component 3

collagen-1 collagen-1

collagen-2 collagen-2

collagen-3 collagen-3

hDF-1 hDF-1

hDF-2 hDF-2

hDF-3 hDF-3

hDF-4 hDF-4

hpsc-1 hpsc-1

hpsc-2 hpsc-2

hpsc-3 hpsc-3

hpsc-4 hpsc-4

pri-1 pri-1

pri-2 pri-2

pri-3 pri-3
