# Supplementary material for: Human iPSC-derived cardiac-specific extracellular matrix scaffolds for cardiomyocyte maturation and post-myocardial infarction repair
Source: Bioact Mater. 2025 Sep 17;55:114–30. doi: 10.1016/j.bioactmat.2025.06.044 (PMC12481509; doi:10.1016/j.bioactmat.2025.06.044)
Supplement: Multimedia component 1 [file mmc1.docx]

**Supplementary methods**

***1. LC-MS Sample Preparation***

100 μg of protein per sample was centrifuged at 12,000 rpm for 10 min at 4°C. Subsequently, 200 μL of 50 mM ammonium bicarbonate was added to the concentrate, followed by centrifugation and repeat once. After being reduced by 10 mM DTT at 56°C for 1 h and alkylated by 20 mM IAA at room temperature in dark for 1h, the sample was centrifuged at 12,000 rpm for 10 min at 4°C and washed once with 50 mM ammonium bicarbonate. Then, 100 μL of 50 mM ammonium bicarbonate and free trypsin at a ratio of 1:50 were added to the protein solution. The solution was incubated at 37°C overnight. Finally, the sample was centrifuged at 12,000 rpm for 10 min at 4°C. Next, 100 μL of 50 mM of ammonium bicarbonate was added to the device, centrifuged, and repeated once. Finally, the extracted peptides were lyophilized to near dryness and resuspended in 20 μL of 0.1% formic acid before LC-MS/MS analysis.

*Nano LC-MS/MS Analysis*

- Nanoflow UPLC: UltiMate™ 3000 RSLCnano System (ThermoFisher Scientific, USA) Nanocolumn: Trapping column (PepMap C18, 100 Å, 100 μm × 2 cm, 5 μm) and analytical column (PepMap C18, 100Å, 75 μm × 50 cm, 2 μm)
- Loaded sample volume: 1 μg
- Mobile phase: A: 0.1% formic acid in water; B: 0.1% formic acid in 80% acetonitrile. Total flow rate: 250 nL/min
- LC linear gradient: from 2 to 8% buffer B in 3 min, from 8% to 20% buffer B in 56 min, from 20% to 40% buffer B in 37 min, then from 40% to 90% buffer B in 4 min.

*Mass Spectrometry*

The full scan was performed between 300 and 1,650 m/z at the resolution of 60,000 at 200 m/z. The automatic gain control target for the full scan was set to 3e6. The MS/MS scan was operated in Top 20 mode using the following settings: resolution 15,000 at 200 m/z; automatic gain control target 1e5; maximum injection time 19ms; normalized collision energy at 28%; isolation window of 1.4 Th; charge sate exclusion: unassigned, 1, > 6; dynamic exclusion 30 sec.

*Data Analysis*

Nine raw MS files were analyzed and searched against the human protein database based on the species of the samples using MaxQuant (1.6.2.14). The parameters were set as follows: the protein modifications were carbamidomethylation (C) (fixed), oxidation (M) (variable); the enzyme specificity was set to trypsin; the maximum missed cleavages were set to 2; the precursor ion mass tolerance was set to 10 ppm, and MS/MS tolerance was 0.6 Da. In this experiment, a total of 700 proteins were identified. Proteins were divided into two categories. Fold-change (FC) > 2 was considered up-regulation, while FC < 0.5 (1/2) was considered down-regulation. The number of differentially expressed proteins (DEPs) was summarized in Table 1. The detailed protein and peptide identification and quantification information has been listed in the supplemental Excel sheets.

**Table 1** Summary of differentially expressed proteins (DEPs)

| **Group Name** | **Up-regulated**  **(FC>2, p<0.05)** | **Down-regulated**  **(FC<1/2, p<0.05)** |
| --- | --- | --- |
| hiPSC-CF vs Pri-CF | 86 | 290 |
| hiPSC-CF vs hDF | 207 | 115 |

***2. RNA-seq analysis***

*Reads mapping to the reference genome.*

Reference genome and gene model annotation files were downloaded from genome website browser (NCBI/UCSC/Ensembl) directly. Indexes of the reference genome were built using STAR and paired-end clean reads were aligned to the reference genome using STAR (v2.5). STAR used the method of Maximal Mappable Prefix (MMP) which can generate a precise mapping result for junction reads.

*Quantification of gene expression level*

STAR counts the number of reads per gene while mapping. The counts coincide with those produced by htseq-count with default parameters. Then, the FPKM of each gene was calculated based on the length of the gene and the reads count mapped to this gene. FPKM, Reads Per Kilobase of exon model per Million mapped reads, considers the effect of sequencing depth and gene length for the reads count at the same time and is currently the most commonly used method for estimating gene expression levels (Mortazavi et al., 2008).

*Differential expression analysis*

(For DESeq2 with biological replicates) Differential expression analysis between two conditions/groups (two biological replicates per condition) was performed using the DESeq2 R package (1.14.1). DESeq2 provides statistical routines for determining differential expression in digital gene expression data using a model based on negative binomial distribution. The resulting P-values were adjusted using Benjamini and Hochberg's approach for controlling the False Discovery Rate (FDR). Genes with an adjusted P-value <0.05 found by DESeq2 were assigned as differentially expressed.

*For edger without biological replicates:* Prior to differential gene expression analysis, for each sequenced library, the read counts were adjusted by edgeR program package through one scaling normalized factor. Differential expression analysis of two conditions was performed using the edgeR R package (3.16.5). The P values were adjusted using the Benjamini & Hochberg method. A corrected P-value of 0.05 and absolute foldchange of 1 were set as the threshold for significantly differential expression. The Venn diagrams were prepared using the function vennDiagram in R based on the gene list for different groups.

***Correlations***

Genes with 0 FPKM are assigned a value of 0.001 to allow for log adjustment. Correlation was determined using the cor.test function in R with options set alternative ="greater" and method = "Spearman".

***Clustering***

To identify the correlation among differences, we clustered different samples based on FPKM expression levels. Clustering was performed using hierarchical clustering, SOM (Self-Organizing Maps), and k-means. To identify the optimal classification, we evaluated the k-means clustering results using the silhouette coefficient. Clustering methods were implemented in R with default parameters unless otherwise specified.

***GO and KEGG enrichment analysis of differentially expressed genes***

Gene Ontology enrichment analysis of differentially expressed genes was implemented by the clusterProfiler R package, in which gene length bias was corrected. GO terms with corrected Pvalue less than 0.05 were considered significantly enriched by differential expressed genes.

KEGG is a database resource for understanding high-level functions and utilities of the biological system, such as the cell, the organism and the ecosystem, from molecular level information, especially large-scale molecular datasets generated by genome sequencing and other high-through put experimental technologies (http://www.genome.jp/kegg/). We used the clusterProfiler R package to test the statistical enrichment of differential expression genes in KEGG pathways.

***PPI analysis of differentially expressed genes***

PPI analysis of differentially expressed genes was based on the STRING database, which contained known and predicted Protein-Protein Interactions. For the species existing in the database (like human and mouse), we constructed the networks by extracting the target gene lists from the database.

***Fusion gene analysis***

Fusion gene refers to the product of two genes where all or part of the sequences fuse, resulting in the hybrid gene, usually caused by reasons such as chromosome translocation and problems. We used Star-fusion (v1.5.0) software analysis and detection of fusion genes.

***Alternative splicing analysis***

Alternative Splicing is an important mechanism for regulating the expression of genes and the variable of protein. rMATS (3.2.5) software was used to analysis the ASevent.

***SNP analysis***

We deal with each sample's bam alignment results using Picard tools (v1.111) and Sam tools (v0.1.18), including reordering, sorting, adding head information, marking duplicates, local realignment around indels, and base quality score recalibration. Then, we call snp using the tool HaplotypeCaller in the GATK4.1 version. Finally, we use Annovar to annotate SNP against the dbSNP database and other databases.

***Differentially expressed gene annotation***

The differentially expressed gene was annotated using TFCat and the COSMIC database. TFCat is a curated catalog of mouse and human transcription factors (TF) based on a reliable core collection of annotations obtained by expert review of scientific literature. COSMIC is a database designed to store and display somatic mutation information and related details, which contains information relating to human cancers.

**Supplementary data**


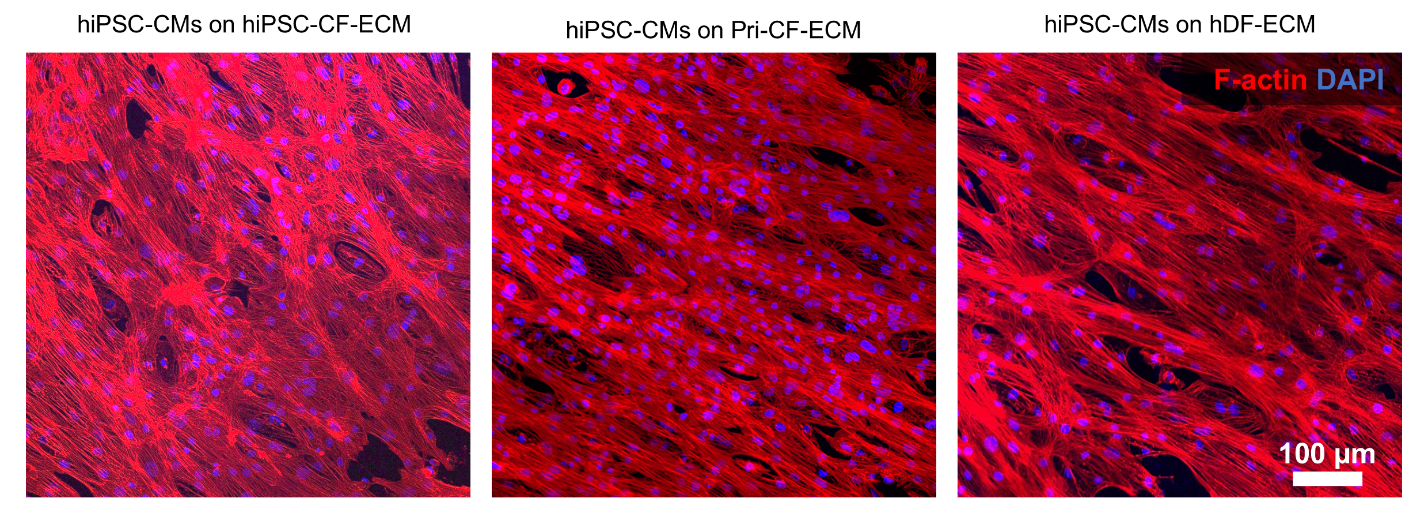


**Figure S1.** hiPSC-CMs cultured on cardiac (hiPSC-CF-ECM and Pri-CF-ECM) and non-cardiac (hDF-ECM) scaffolds organized in an anisotropic architecture following aligned ECM fiber deposition (F-actin (red), DAPI (blue)).


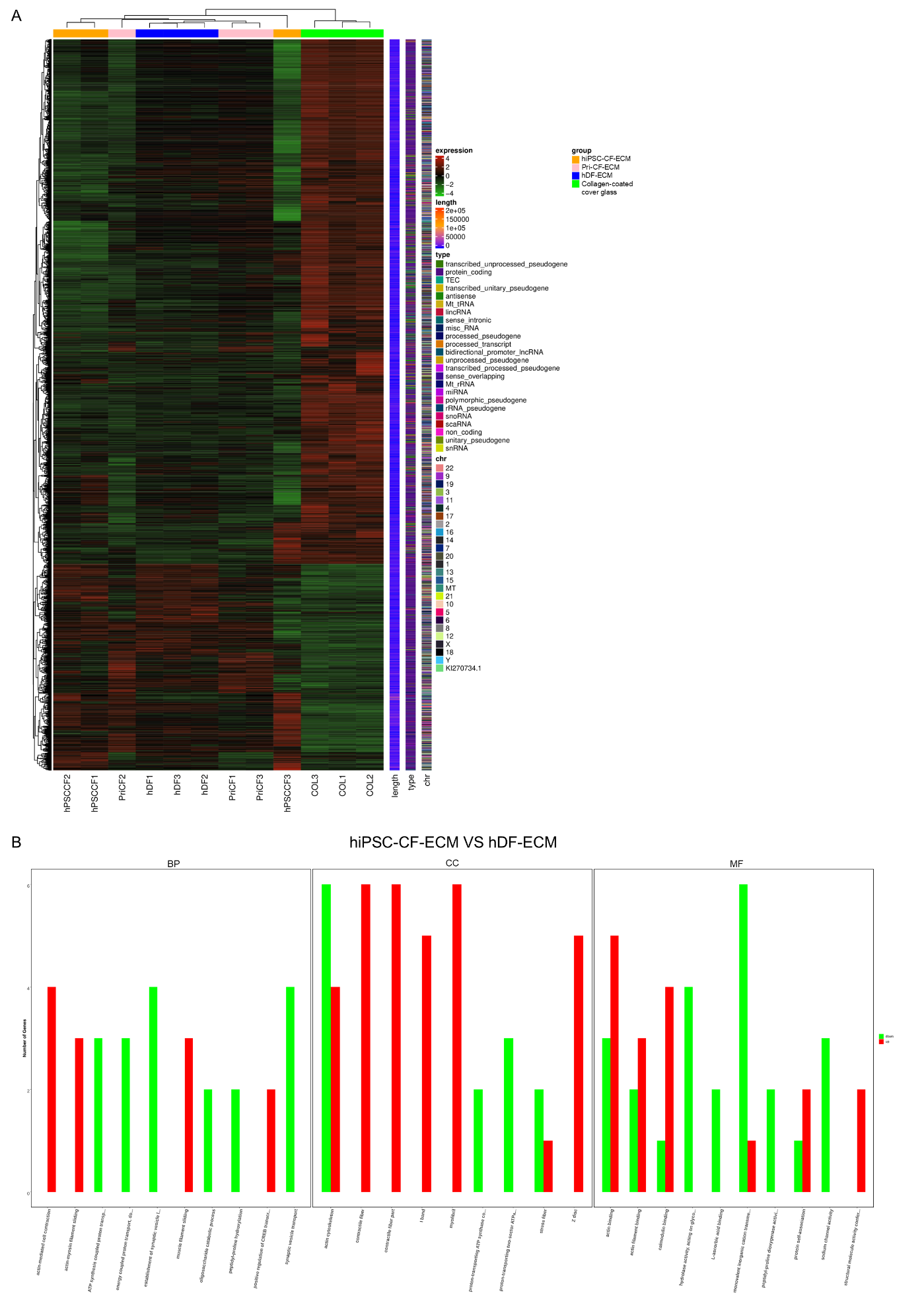


**Figure S2.** **(A)** Hierarchical clustering of hiPSC-CMs cultured on hiPSC-CF-ECM, Pri-CF-ECM, hDF-ECM, and collagen-I based on mRNA expression obtained from RNA-seq. Heatmap indicates the differentially expressed genes (DEGs) from hiPSC-CMs cultured of various ECM constructs and compared with control collagen-I coated surface. The color in each grid does not reflect the gene expression value, but the value obtained after homogenizing the expression data rows. The chromosome to which each gene belongs, the gene's length, and the biological type of the gene are also added to the heatmap. **(B)** Gene ontology (GO) enrichment analysis reveals up/ downregulation of cardiac-specific structural and functional genes activated in hiPSC-CMs cultured on hiPSC-CF-ECM Vs. hDF-ECM. The abscissa in the figure is GO Term, and the ordinate is GO Term The level of significance of the set.


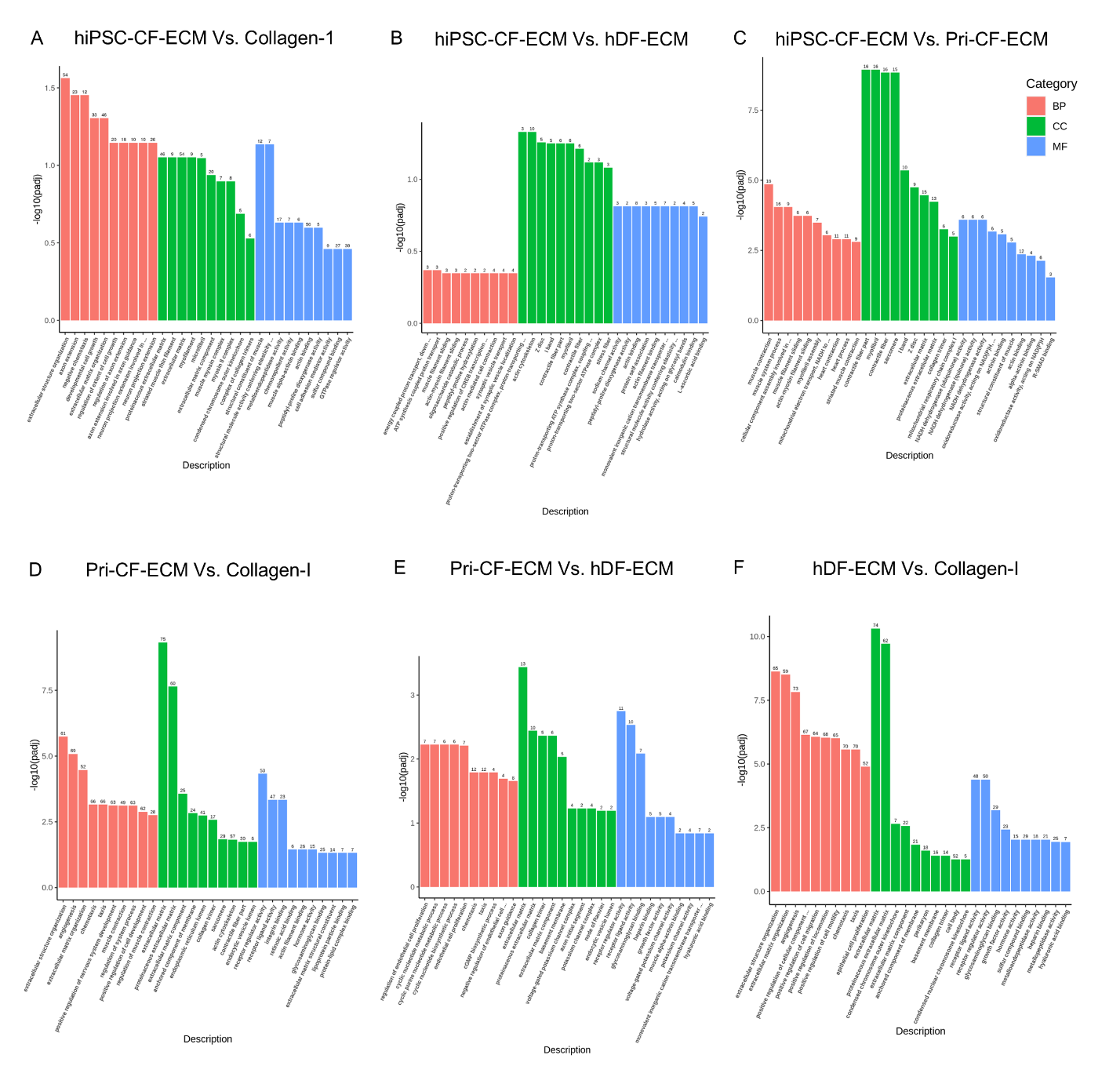


**Figure S3.** GO enrichment analysis histogram comparing the expression profile in hiPSC CMs cultured on (A) hiPSC-CF-ECM Vs. Collagen-I, (B) hiPSC-CF-ECM Vs. hDF-ECM, (C) hiPSC-CF-ECM Vs. Pri-CF-ECM, (D) Pri-CF-ECM Vs. Collagen-I, (E) Pri-CF-ECM Vs. hDF-ECM and (F) hDF-ECM Vs. Collagen-I. The abscissa in the figure is GO Term, and the ordinate is GO Term's level of significance of enrichment, expressed as -log10 (padj). Different colors represent different functional categories.


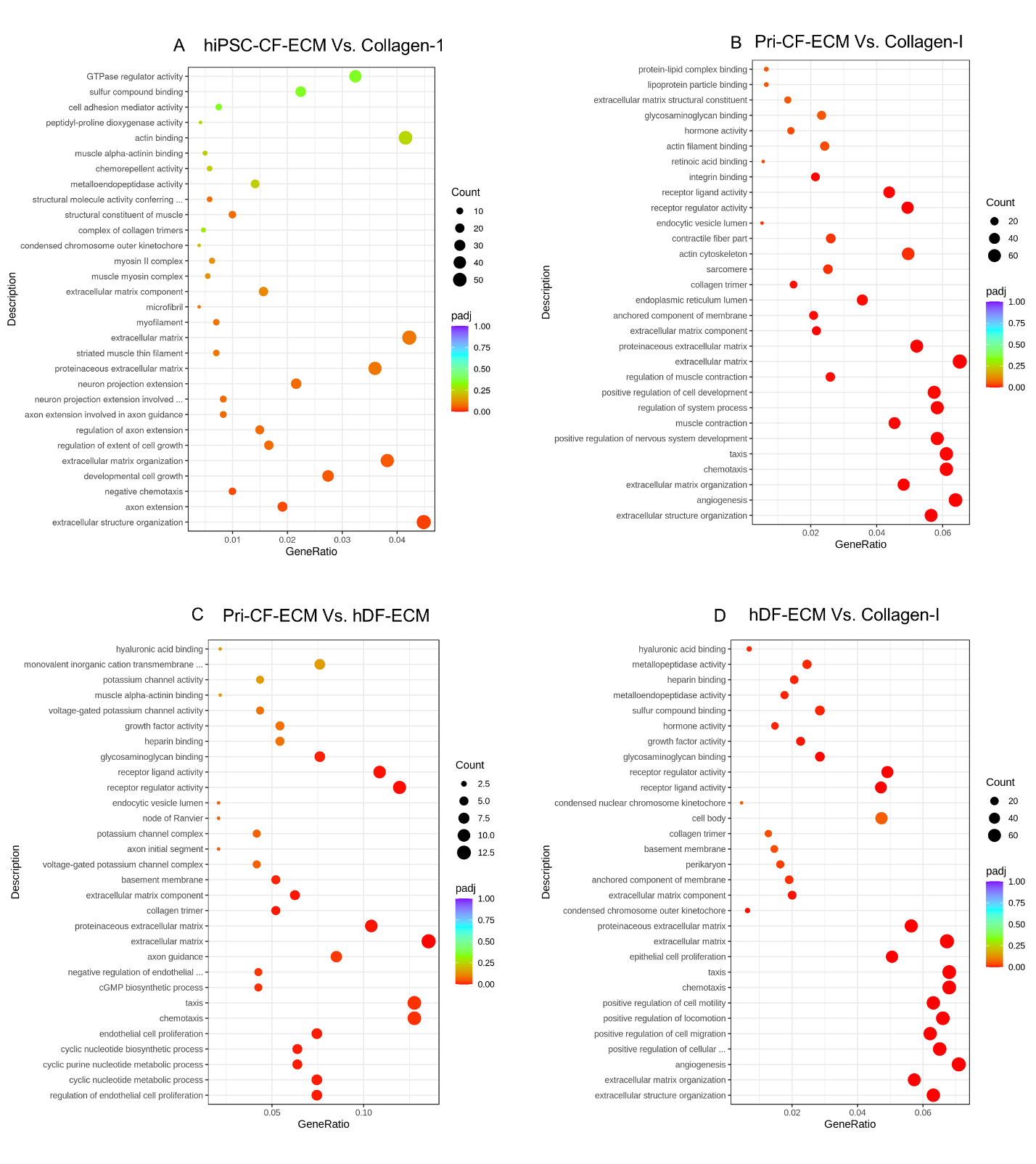


**Figure S4** GO Enrichment Analysis Scatter Plot comparing the expression profile in hiPSC CMs cultured on (A) hiPSC-CF-ECM Vs. Collagen-I, (B) Pri-CF-ECM Vs. Collagen-I, (C) Pri-CF-ECM Vs. hDF-ECM and (C) hDF-ECM Vs. Collagen-I. The abscissa in the graph is the ratio of the differential gene number to the total number of differential genes on the GO Term, and the ordinate is GO Term.


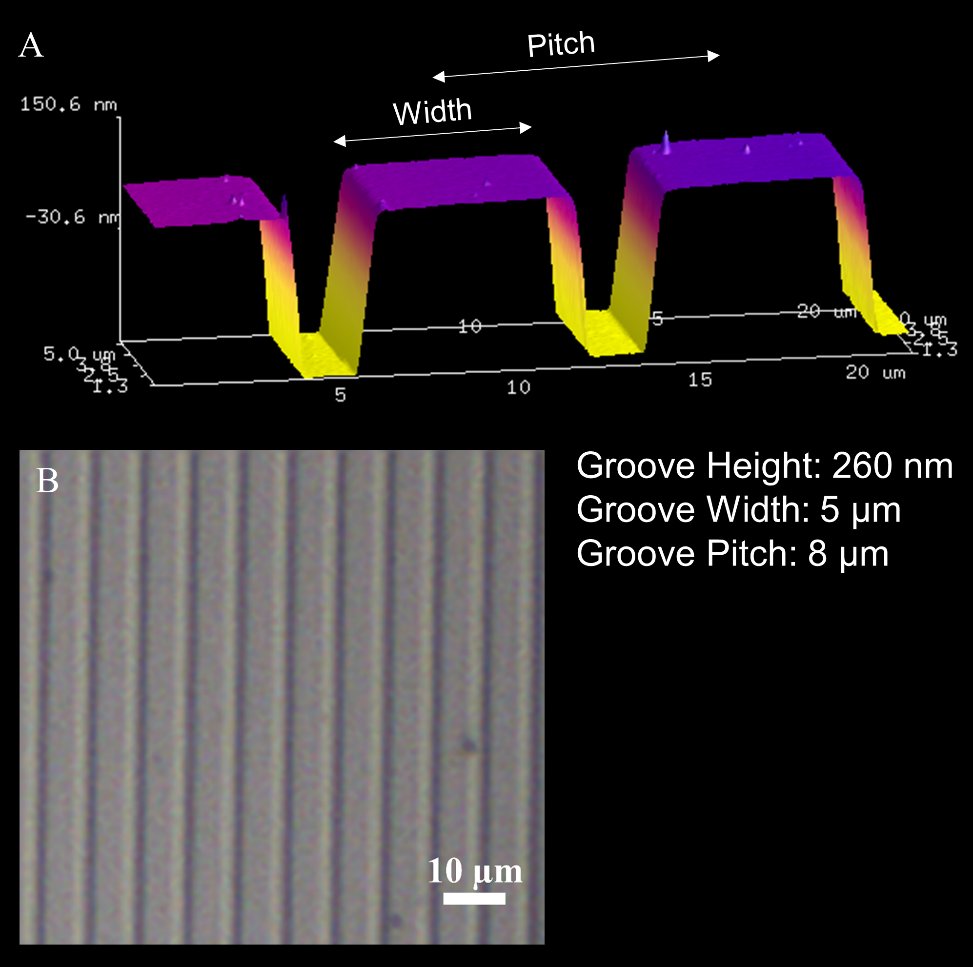


**Figure S5 Characterization of micro-grated PDMS using atomic force microscopy (AFM).** (A)Polydimethylsiloxane (PDMS) molds with aligned grooves were cast from micro-grated silicon wafers with 5 µm grating width, 8 µm grating pitch, and 260 nm grating depth as measured by AFM. (B) Image of the micro-grated PDMS captured by AFM-inbuilt camera.

**
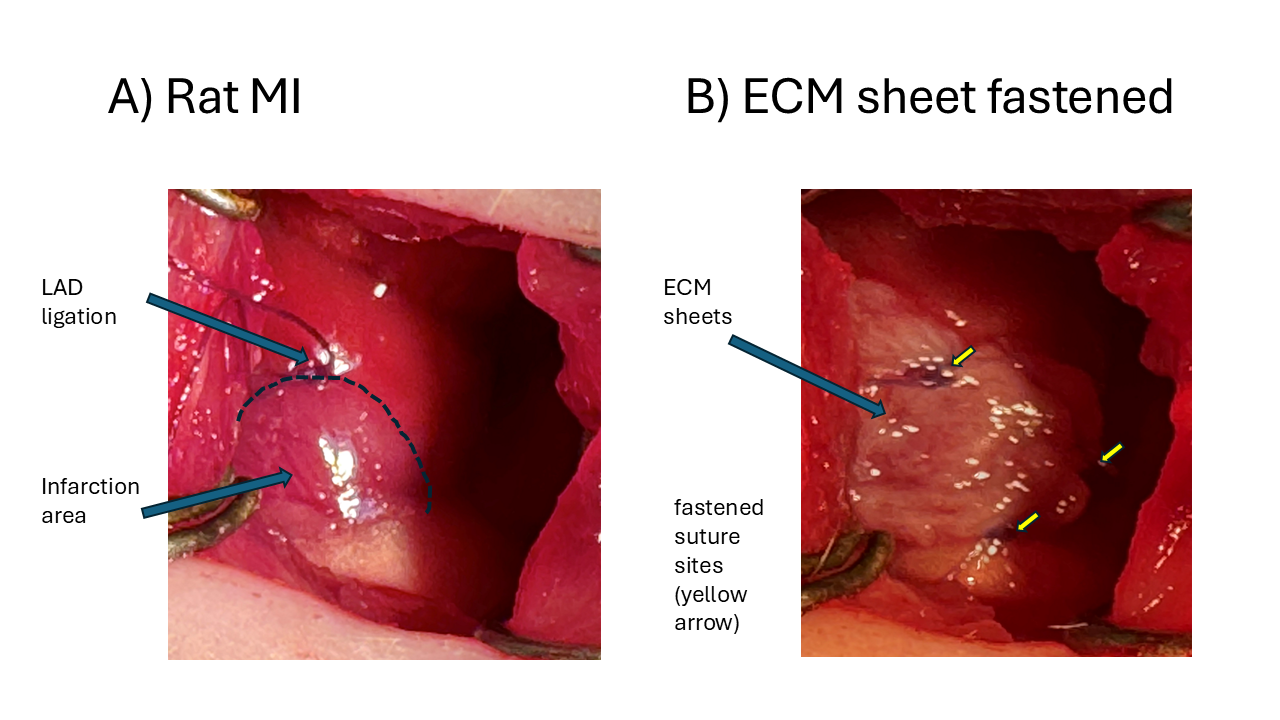
**

**Figure S6 Intraoperative scene of ECM sheets being sutured to the rat heart.** (A) Infarct area following LAD ligation and (B) ECM sheet implantation.


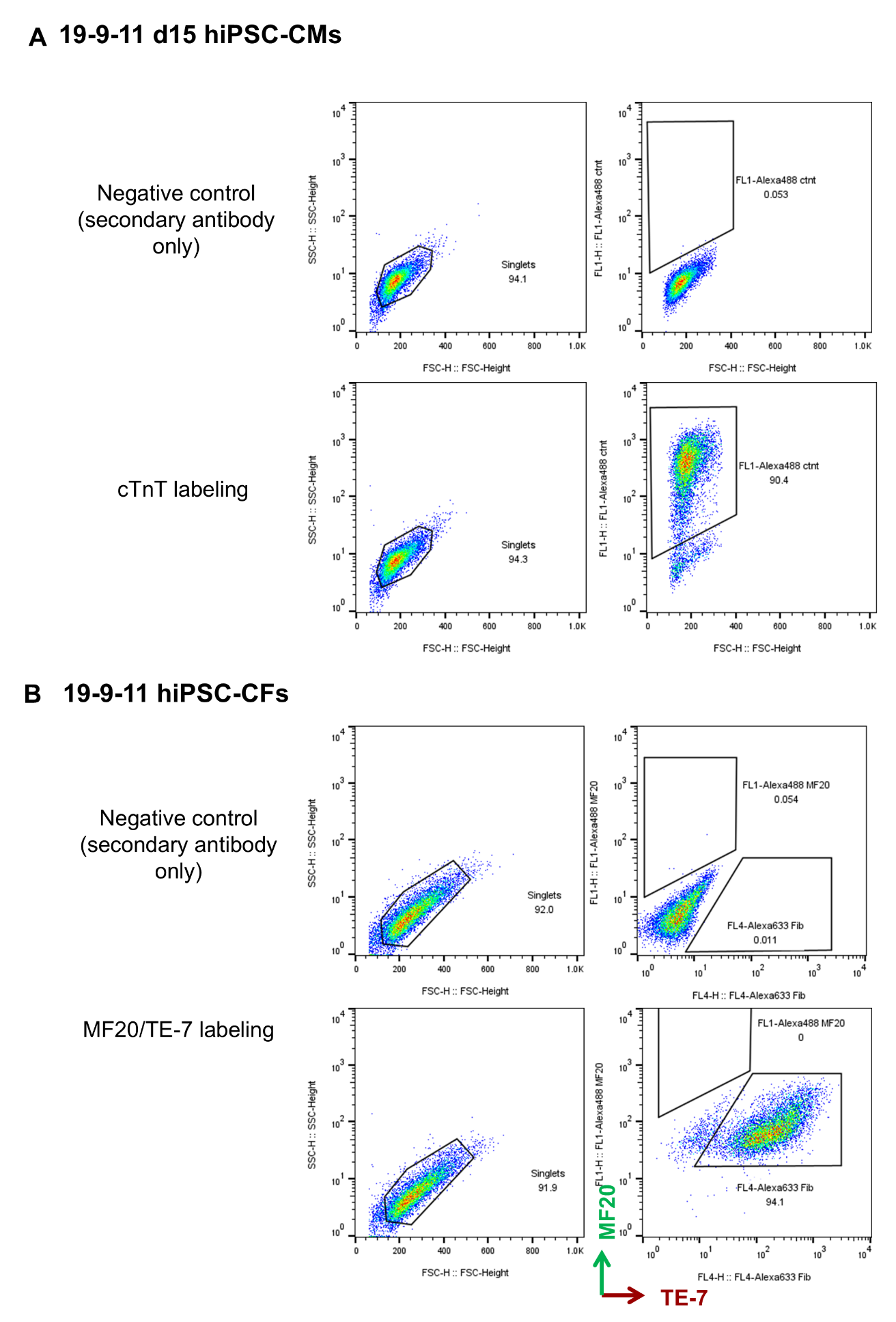


**Figure S7. Flow cytometry gating for hiPSC-CMs and hiPSC-CF quantification.** (A) Dot plots of flow cytometry gating for 19-9-11 day 15 hiPSC-CMs preparation, immunolabeled for cardiac troponin T (cTnT). The upper panels show a negative control with secondary antibody only to generate gates. Left panels show a selection of singlets for analysis in the right panels. 90.4% of the cells are positive for cTnT. (B) Dot plots of flow cytometry gating for 19-9-11 hiPSC-CFs preparation, dual immunolabeled for sarcomeric myosin (MF20) and the fibroblast-specific antibody TE7. Upper panels show a negative control with secondary antibodies only to generate gates. Left panels show a selection of singlets for analysis in the right panels. 94.1% of the cells are positive for the fibroblast marker TE-7.

**Supplementary table 1A:**

| Freq. | Conditions | CV_L_   (cm/s) | CV_T_  (cm/s) | AR (CV_L_/CV_T_) | APD20% (ms) | APD50% (ms) | APD80% (ms) |
| --- | --- | --- | --- | --- | --- | --- | --- |
| 1 Hz | hiPSC-CF | 31.5 ± 3.8* | 23.0 ± 5.7 | 1.41 ± 0.22^#^ | 197 ± 3* | 319 ± 16 | 361 ± 18 |
|  | Pri-CF | 35.5 ± 5.2* | 26.3 ± 3.2 | 1.37 ± 0.30 | 192 ± 14* | 298 ± 17 | 339 ± 14 |
|  | hDF | 31.4 ± 6.2* | 26.4 ± 5.7 | 1.20 ± 0.19 | 205 ± 42* | 335 ± 75* | 372 ± 76 |
|  | Collagen-I | 21.6 ± 0.6 | 20.0 ± 0.4 | 1.08 ± 0.05 | 138 ± 11 | 242 ± 15 | 290 ± 11 |
| 2 Hz | hiPSC-CF | 25.6 ± 5.0 | 16.9 ± 6.2 | 1.62 ± 0.37^#^ | 173 ± 17* | 251 ± 18* | 287 ± 17* |
|  | Pri-CF | 25.1 ± 8.0 | 17.0 ± 5.2 | 1.51 ± 0.41 | 158 ± 9 | 230 ± 18 | 266 ± 16 |
|  | hDF | 22.2 ± 6.4 | 20.7 ± 5.1 | 1.07 ± 0.13 | 170 ± 9* | 251 ± 14* | 290 ± 12* |
|  | Collagen-I | 14.5 ± 3.7 | 13.6 ± 3.3 | 1.06 ± 0.04 | 136 ± 12 | 214 ± 17 | 253 ± 19 |
| 3 Hz | hiPSC-CF | 20.8 ± 4.1* | 13.6 ± 5.9 | 1.62 ± 0.34 | 120 ± 6 | 173 ± 15 | 207 ± 12 |
|  | Pri-CF | 19.0 ± 3.2 | 13.4 ± 3.1 | 1.43 ± 0.14^#^ | 112 ± 3 | 169 ± 24 | 202 ± 22 |
|  | hDF | 21.3 ± 1.1* | 16.4 ± 0.8 | 1.30 ± 0.01^#^ | 118 ± 11 | 182 ± 20 | 215 ± 18 |
|  | Collagen-I | 10.8 ± 4.3 | 10.4 ± 4.3 | 1.08 ± 0.08 | 112 ± 17 | 169 ± 22 | 207 ± 21 |

CV_L_ and CV_T_ - longitudinal and transversal conduction velocity; AR - anisotropy ratio; APD - action potential decay duration at x% of repolarization. *P* values were determined by one-way ANOVA with multiple comparisons to Collagen-I (if significant marked as *) and by one-sample t-test compere mean to 1 (if significant marked as ^#^). Data represented as mean ± SD.

**Supplementary table 2: Comparison of cardiac ECM composition.**

|  | **LC-MS Relative Abundance** | | | | | | | | |
| --- | --- | --- | --- | --- | --- | --- | --- | --- | --- |
| **Cardiac ECM Source** | **Fibronectin** | **Fibrillin-1** | **Fibrillin-2** | **HSPG2** | **Periostin** | **Collagen 1** | **Collagen 3** | **Collagen 4** | **Collagen 6** |
| Fetal Rat [89] | 37% | 11% | 7% | ? | ? | 34% | ? | ? | 3% |
| Fetal mouse [23] | 26% | 13% | 8% | 12% | 7% | 11% | ? | 8% | 11% |
| Adult mouse [23] | 4% | 18% | 0% | 8% | 1% | 38% | 4% | 5% | 4% |
| Adult human [29] | 7% | 7% | 4% | 3% | ? | 28% | 8% | 15% | 10% |
| hiPSC-CF | 22.51% | 3.41% | 0.8% | 1.74% | 2.61% | 0.97% | 0.033% | 0.54% | 30.03% |
| Pri-CF | 58.39% | 9.03% | 0.175% | 10.13% | 0.64% | 0.189% | 0.028% | 0.28% | 8.98% |
| hDF | 23.00% | 9.29% | 0.23% | 1.94% | 1.76% | 0.466% | 0.042% | 0.00055% | 29.32% |
